# Global remodeling of nucleosome positions in *C*. *elegans*

**DOI:** 10.1186/1471-2164-14-284

**Published:** 2013-04-26

**Authors:** George Locke, Devorah Haberman, Steven M Johnson, Alexandre V Morozov

**Affiliations:** 1 Department of Physics and Astronomy and BioMaPS Institute for Quantitative Biology, Rutgers University, Piscataway, NJ, 08854, USA; 2 Department of Chemistry, Princeton University, Princeton, NJ, 08544, USA; 3 Department of Microbiology and Molecular Biology, Brigham Young University, Provo, UT, 84602, USA

**Keywords:** Nucleosome, Histone-DNA interactions, Chromatin domains, Nucleosome positioning

## Abstract

**Background:**

Eukaryotic chromatin architecture is affected by intrinsic histone-DNA sequence preferences, steric exclusion between nucleosome particles, formation of higher-order structures, and *in vivo* activity of chromatin remodeling enzymes.

**Results:**

To disentangle sequence-dependent nucleosome positioning from the other factors, we have created two high-throughput maps of nucleosomes assembled *in vitro* on genomic DNA from the nematode worm *Caenorhabditis elegans*. A comparison of *in vitro* nucleosome positions with those observed in a mixed-stage, mixed-tissue population of *C*. *elegans* cells reveals that *in vivo* sequence preferences are modified on the genomic scale. Indeed, G/C dinucleotides are predicted to be most favorable for nucleosome formation *in vitro* but not *in vivo*. Nucleosome sequence read coverage *in vivo* is distinctly lower in chromosome arms than in central regions; the observed changes in apparent nucleosome sequence specificity, likely due to genome-wide chromatin remodeler activity, contribute to the formation of these megabase-scale chromatin domains. We also observe that the majority of well-positioned *in vivo* nucleosomes do not occupy thermodynamically favorable sequences observed *in vitro*. Finally, we find that exons are intrinsically more amenable to nucleosome formation compared to introns. Nucleosome occupancy of introns and exons consistently increases with G/C content *in vitro* but not *in vivo*, in agreement with our observation that G/C dinucleotide enrichment does not strongly promote *in vivo* nucleosome formation.

**Conclusions:**

Our findings highlight the importance of both sequence specificity and active nucleosome repositioning in creating large-scale chromatin domains, and the antagonistic roles of intrinsic sequence preferences and chromatin remodelers in *C*. *elegans*.

Sequence read data has been deposited into Sequence Read Archive (http://www.ncbi.nlm.nih.gov/sra; accession number SRA050182). Additional data, software and computational predictions are available on the Nucleosome Explorer website (http://nucleosome.rutgers.edu).

## Background

In eukaryotes, genomic DNA is packaged into chromatin [1,2]. DNA in the chromatin state is wrapped around consecutive histone octamers, creating a “beads-on-a-string” fiber which is subsequently folded into higher-order structures [3,4]. The fundamental unit of chromatin is the nucleosome core particle – 147 base pairs (bps) of DNA wrapped in a left-handed superhelix around each histone octamer [5,6]. Nucleosome positioning strongly affects gene regulation and other vital functions such as cell replication and DNA repair, both by occluding functional elements (transcription factor binding sites, splicing signals, etc.) and by recruiting chromatin remodeling, regulatory and transcriptional machinery through association with histones and nucleosome-packaged DNA [7-9]. The strength of this association is often modulated by a combinatorial array of post-translational histone tail modifications, including acetylation, methylation, ubiquitination and phosphorylation [10-12]. Thus understanding the relative role of various factors that define nucleosome positions in living cells is a challenging task.

One of these factors is DNA itself – direct measurements of histone-DNA binding affinities have established that changes to DNA sequence can vary the energy of nucleosome formation by as much as 2–3 kcal/mol, although typical differences in energy between two randomly picked genomic nucleosomal sequences are expected to be smaller [13-19]. These studies have also probed the rules that control histone-DNA binding affinity, focusing in particular on the 10–11 bp periodic dinucleotide motifs commonly found in high-affinity nucleosomal sequences [14,17]. The 10–11 bp periodicity helps minimize the cost of bending a 147 bp-long DNA molecule into the nucleosomal superhelix by placing G/C dinucleotides where the major groove is compressed (i.e., the minor groove faces away from the surface of the histone octamer) and A/T dinucleotides where the minor groove is compressed (i.e., faces toward the histone octamer) [1,14,20,21]. More recently, genome-wide maps of nucleosome positions have revealed that nucleosomes simply tend to occupy G/C-enriched and A/T-depleted sites [22-25]. The G/C dinucleotide content in stable nucleosomes reconstituted *in vitro* increases towards the dyad, and was found to be elevated in nucleosomes and depleted in linkers in several *in vitro* and *in vivo* assays [22,25-27]. The relative contribution of increased G/C content vs. 10–11 bp periodic motifs to the free energy of nucleosome formation is currently unknown. It is possible that the MNase-based experimental procedure used to isolate mononucleosomes leads to enrichment in GC content [28]. However, there is a reason to believe that G/C mono- and di-nucleotides do promote nucleosome formation and are not simply an artifact of the experimental setup [29].

Although sequence dependence of histone-DNA binding affinity is well established, its relative importance in shaping chromatin architecture of living cells has proven more controversial [20,27]. Since free energies required to displace a nucleosome are of the order of several kcal/mol, they are readily available in the cell, for example through the process of ATP hydrolysis utilized by a number of ATP-dependent chromatin-remodeling enzymes [30-33]. Nucleosome positions can also be modified *in vivo* through chromatin fiber formation which tends to constrain linker DNA lengths [34,35], and through direct competition with non-histone DNA-binding factors. Previous studies in *Saccharomyces cerevisae* have compared chromatin assembled *in vitro* on genomic DNA fragments (where, apart from potential subtle effects related to folding into higher-order structures, nucleosome positions are dictated solely by intrinsic sequence preferences and steric exclusion) with chromatin extracted from living cells [22,26,27]. These studies have shown that although bulk nucleosomes are not strongly sequence-specific, models trained on *in vitro* data can predict a subset of *in vivo* nucleosomes occupying intrinsically favorable sites. It is unclear to what extent this conclusion can be transferred to complex metazoan organisms with multiple cell types, where nucleosome occupancy may be more strongly affected by chromatin remodelers and formation of large-scale chromosomal domains. Furthermore, nucleosome positioning is thought to play a major role in directing co-transcriptional splicing by demarcating metazoan exon-intron architecture [36,37]. Implementing this function may entail overwriting intrinsic histone-DNA sequence preferences.

To clarify these issues, we have carried out *in vitro* nucleosome assembly on genomic DNA from the nematode worm *Caenorhabditis elegans*. High-throughput sequencing was used to create large-scale maps of *in vitro* nucleosome positions. The *in vitro* maps were compared with two maps of nucleosome positions in a mixed-stage, mixed-tissue population of *C*. *elegans* cells previously obtained by high-throughput Illumina and SOLiD sequencing [38,39], and with three nucleosome maps in *C*. *elegans* embryonic cells, adult somatic cells, and a mix of adult somatic and germ cells obtained by another group using paired-end Illumina sequencing [40]. These comprehensive high-resolution maps have enabled us to carry out a detailed analysis of deviations from intrinsic nucleosome positioning in living cells of a metazoan organism. Our results suggest that chromatin remodelers operating at the level of single nucleosomes play a role in the formation of large-scale chromatin domains that make *C*. *elegans* autosome termini distinct from central regions.

## Results and discussion

### *In vitro* and *in vivo* nucleosome maps

In order to focus on the contribution that primary DNA sequence has on nucleosome positioning and formation, we carefully chose our genomic template and reconstitution conditions for the *in vitro* nucleosome experiments. To create *in vitro* nucleosome positioning maps, high molecular weight *C*. *elegans* genomic DNA was digested into fragments by either Hinc II or Rsa I restriction enzyme and reconstituted into nucleosomes using recombinant histone octamers (Methods). In order to minimize potential biases due to end effects of short DNA fragments and steric hindrances due to neighboring nucleosome formation [41], nucleosomes were reconstituted on a well-defined restriction set of DNA fragments and at a high DNA-to-histone mass ratio. Assembled nucleosomes were treated with micrococcal nuclease (MNase) and the mononucleosomal DNA was isolated and sequenced on an Illumina high-throughput sequence analyzer (Methods). The sequence reads were mapped to the WS190 *C*. *elegans* genome, creating two independent *in vitro* nucleosome coverage profiles which we will refer to as Hinc II and Rsa I maps, respectively. We observe that Rsa I and Hinc II sequence reads are enriched in the vicinity of their respective cut sites (as seen in Additional file 1: Figure S1, which shows average read counts near cut sites). This end effect is not observed *in vivo* (data not shown), and has a potential to introduce a bias into subsequent analysis of *in vitro* nucleosomal maps. The bias can be controlled by excluding the neighborhood around each cut site from further analysis, although the fraction of bps removed via this filter may be substantial depending on the neighborhood size (Methods). Nucleosome coordinates in the two *in vivo* maps produced by Fire and collaborators (referred to collectively as our *in vivo* maps or individually as Gu & Fire and Valouev et al. maps hereafter) are as previously reported [38,39]. Nucleosome sequence reads from Ercan et al. [40] (extracted from embryos, adults and germlineless adults) were mapped as described in Methods.

### Nucleosome distribution over large-scale chromosomal domains

**Figure 1:**
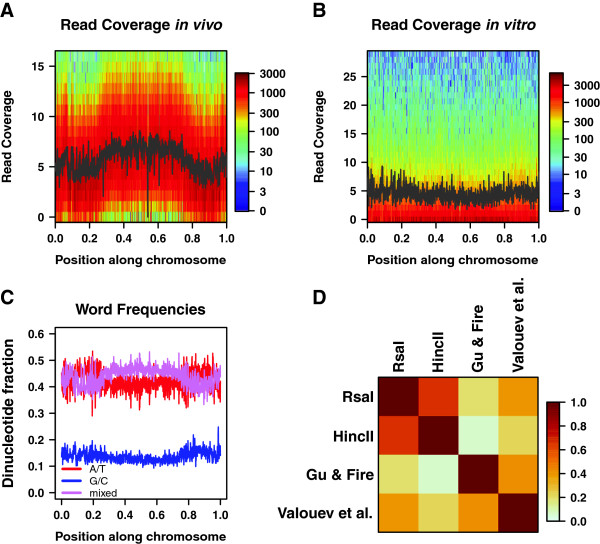
**Global distribution of nucleosomal reads. A)** Two-dimensional histogram of read coverage *in vivo*. Chromosome III was divided into one thousand segments of equal length. The relative position of each segment is shown on the x-axis. The nucleosome read coverage, as measured *in vivo* by Gu & Fire [38], is shown on the y-axis. For each segment, a color-coded histogram shows the number of bases with a given read coverage. The black line shows mean read coverage in each segment. The range of the y-axis excludes the top 1.0% of bases with the highest read coverage. **B)** Two-dimensional histogram of read coverage *in vitro*. Same as (A), but with nucleosome read coverage data from the Hinc II *in vitro* assay. **C)** Dinucleotide frequencies. Chromosome III was divided into one thousand segments of equal length as in (A), and the dinucleotide composition in each segment was plotted against its relative genomic coordinate. A/T dinucleotides, composed only of A and T, are shown in red, G/C dinucleotides, composed only of G and C, are shown in blue, and mixed dinucleotides, including one A or T and one G or C nucleotide, are shown in pink. **D)***In vitro* and *in vivo* nucleosome maps. Pearson correlations between read coverage profiles from each indicated experiment are plotted. For each comparison, a single correlation coefficient is calculated across all chromosomes.

We find that nucleosome sequence reads in both of our *in vivo C*. *elegans* maps are arranged in a distinct global pattern – for each chromosome, the number of reads is highest in the central domain and gradually decreases towards the ends of the chromosome. Thus chromosomal arms are depleted of nucleosome reads relative to the central domain (Figure 1A, Additional file 1: Figure S2 and Figure S3). The read coverage difference is more pronounced in autosomes but is also clearly visible in the X chromosome (Additional file 1: Figure S2 and Figure S3). One possible explanation is that *in vivo* nucleosome coverage is indeed lower in the arms. Another possibility is the differential recovery of sequence reads in the arms vs. central regions caused by distinct patterns of post-translational modifications, chromatin tertiary structure and histone variant usage that could affect the efficiency of MNase in producing mononucleosome fragments. In either case, our observations point to substantial differences in chromatin structure of arms and central regions. This depletion of sequence reads from chromosomal arms is not observed *in vitro*, where both Rsa I and Hinc II maps yield flat distributions (Figure 1B, Additional file 1: Figure S2 and Figure S4). The pronounced depletion of sequence reads from terminal domains cannot be explained by the over-representation of repetitive sequences on the chromosomal arms in *C*. *elegans*[42], which would cause fewer nucleosomes to be mapped to such repetitive sequences both *in vitro* and *in vivo*. Moreover, the enrichment over the central region is not observed in a nucleosome-free control experiment in which *C*. *elegans* genomic DNA was digested by MNase [40] (Additional file 1: Figure S2). The maximum number of mismatches allowed when sequence reads are mapped to the reference genome also does not appear to play a role since it was different in the two *in vivo* maps. Further, the *in vitro* coverage profiles remained flat when the maximum number of mismatches permitted in mapping the reads was reduced from one to zero.

The peaked distribution of mapped nucleosomes occurs in two independent experiments utilizing different sequencing platforms: Applied Biosystems SOLiD [39] and Illumina [38], although it is much less pronounced in an older, low-coverage map which utilized the pyrosequencing-based 454 platform [43] (Additional file 1: Figure S2). However, this feature is absent in three nucleosome maps from Ercan et al. [40], which instead exhibit flat, *in vitro*-like profiles (Additional file 1: Figure S2). Although we do not have a definitive explanation for this discrepancy, we note substantial differences between our experimental protocols: Ercan et al. use high-salt (137 mM NaCl) buffer to isolate and freeze the worms, whereas we use low-salt (15 mM NaCl) buffer which is closer to the sodium levels in living cells. Furthermore, Ercan et al. employ formaldehyde crosslinking which could potentially alter chromatin structure. Finally, they use MNase digestion buffer with 110 mM NaCl vs. 15 mM NaCl in our *in vivo* experiments. This order-of-magnitude increase in sodium levels both before and after crosslinking could have an effect on nucleosome positions, as higher salt concentrations are known to shift and re-equilibrate nucleosomes [28]. In any event, these characteristic patterns of nucleosome read coverage appear to be unique to *C*. *elegans*, and are not observed in *S*. *cerevisiae* and human chromatin (Additional file 1: Figure S5).

Our observations are consistent with a study by Ikegami et al. which identified genomic regions that bind to the antibody of the nuclear transmembrane protein LEM-2 [44]. LEM-2, a member of the lamina network, is localized to the nuclear membrane in *C*. *elegans* cells, providing an anchor by which chromosomes are attached to the nuclear envelope [45,46]. Ikegami et al. have shown that in *C*. *elegans* autosomes arms, but not central regions are enriched in LEM-2 and are thus associated with the nuclear lamina while only the left end of the X chromosome is attached to the nuclear membrane. Broad patterns of post-translational histone tail modifications also demarcate central and distal regions, indicating that their chromatin states are significantly different [47]. The arms also show higher meiotic recombination rates than the central domains [48], whereas highly expressed and essential genes tend to be concentrated closer to the center of each chromosome [42,49]. Finally, periodic clusters of A and T nucleotides occur much more frequently in the arms [50]. Thus, chromosome arms and centers form distinct chromatin domains. The marked difference in the nucleosome read coverage between central and distal regions of the autosomes is in agreement with these earlier findings.

### Universal sequence signatures of *in vitro* nucleosome occupancy

We have studied genome-wide correlations between four nucleosome occupancy profiles: two *in vitro*, Rsa I and Hinc II, and two *in vivo*, Gu & Fire and Valouev et al. (Figure 1D). We have found that, if the immediate neighborhoods of Rsa I and Hinc II restriction enzyme cut sites are excluded from the comparison (Methods), the resulting occupancy profiles of the two *in vitro* experiments are much closer to each other (r = 0.66) than to either *in vivo* profile. The observed “end effect” is sizable in this case: without the cut site filter, the correlation decreases to 0.54. Profiles from the two *in vivo* experiments are also closest to each other (r = 0.41) and substantially different from both *in vitro* experiments, although the Valouev et al. nucleosome map is also significantly correlated with the Rsa I *in vitro* profile (r = 0.38). We have also compared our occupancy profiles with four datasets from Ercan et al.: three nucleosome maps (embryos, adults and germlineless adults) and another map in which MNase was used to digest nucleosome-free DNA [40]. As shown in Additional file 1: Figure S6A, Ercan et al. nucleosome maps are most strongly correlated with one another and, surprisingly, with the nucleosome-free control experiment.

In order to study the interplay between sequence-specific nucleosome positioning and observed global patterns of read coverage, we have fitted *N* = *2* position-independent models to all seven genome-wide maps (Methods). The models assign effective nucleosome formation energies to each genomic bp *i* on the basis of the number of mono- and dinucleotides in the 147 bp window covered by a nucleosome that starts at that bp. The models are called position-independent because they consider mono- and dinucleotide counts regardless of their locations within the nucleosomal site. Position-independent models have been shown to reproduce nucleosome occupancy profiles as efficiently as much more complex models that also take 10–11 bp dinucleotide periodicities into account [22]. The fitting parameters of the models can be rigorously interpreted as mono- and dinucleotide energies under the assumption that nucleosome positioning is affected solely by steric exclusion and intrinsic histone-DNA sequence preferences [22,35,51]. While we expect this to be true in our *in vitro* experiments (apart from the effects of chromatin fiber formation which tends to arrange nucleosomes in regular arrays, resulting in preferences for 10–11 bp discretized linker lengths [34,35,51]), nucleosome positions *in vivo* are also affected by chromatin remodeling enzymes and competition with other DNA-binding proteins. Under these conditions, nucleosome formation energies in our models are best understood as scores that reflect both intrinsic sequence preferences and *in vivo* repositioning.

**Figure 2:**
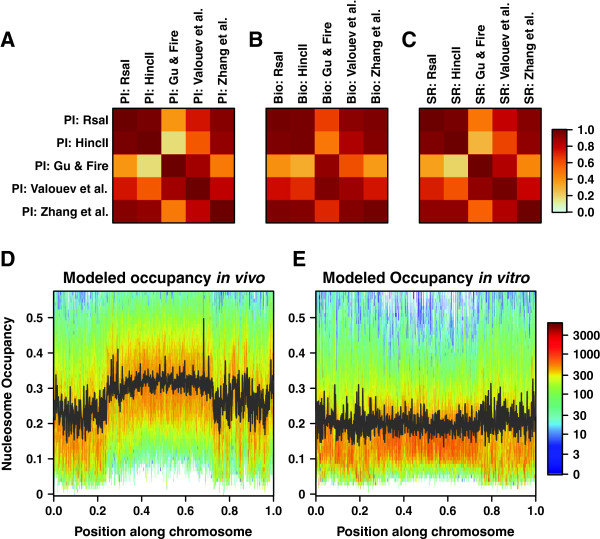
**Sequence-specific models of nucleosome occupancy. A)** Position-independent models. *N* = *2* position-independent (PI) models fit on the datasets indicated were used to predict nucleosome occupancy profiles on the *C. elegans* genome. Pearson correlations between predicted occupancy profiles are plotted. For each comparison, a single correlation coefficient is calculated across all chromosomes. **B)** Bioinformatics versus position-independent models. Same as (A), but comparing predictions made by bioinformatics models (Bio) to those made by position-independent models. **C)** Spatially resolved versus position-independent models. Same as (A), but comparing predictions made by spatially resolved models (SR) to those made by *N = 2* position-independent models. **D)** Two-dimensional histogram of nucleosome occupancy predicted by a position-independent *in vivo* model. Chromosome III was divided into one thousand segments of equal length. The relative position of each segment is shown on the x-axis. The nucleosome occupancy, as predicted by the *N = 2* position-independent model fit on Gu & Fire *in vivo* data [38], is shown on the y-axis. The predicted occupancy was binned into 100 equal intervals in the [0,1] range. For each segment, a color-coded histogram shows the number of bases with a given occupancy. The black line shows mean predicted occupancy in each segment. The range of the y-axis excludes the top 1.0% of bases with the highest occupancy. **E)** Two-dimensional histogram of nucleosome occupancy predicted by a position-independent *in vitro* model. Same as (D), but with occupancy predicted by the *N = 2* position-independent model fit on *in vitro* data from the Hinc II assay.

We find that models trained on *in vitro* nucleosome maps yield very similar dinucleotide rankings. Table 1 shows how our models rank the contributions of each dinucleotide to the total nucleosome formation energy, and it is readily apparent that both *in vitro* models are highly correlated, with A/T dinucleotides least favorable and G/C dinucleotides most favorable. Thus models fit on *in vitro* data predict a canonical positioning mechanism in which G/C dinucleotides are enriched in nucleosomes and A/T dinucleotides are enriched in linkers [22-24]. The strong similarity between *in vitro C*. *elegans* models and our previously published “Zhang et al.” *in vitro* model trained on yeast DNA [22,27] (Table 1, Figure 2A) indicates that our computational approach can be used to extract universal sequence determinants of nucleosome occupancy regardless of the origin of DNA used in the nucleosome assembly experiments. Predicted model parameters are unaffected by the preference for *in vitro* nucleosome assembly in the vicinity of restriction enzyme cut sites (Additional file 1: Figure S1) – a model fit only on Rsa I restriction fragments longer than 2000 bp, which excludes about 86% of the genome, has a 0.91 rank correlation of fitted parameters compared with our standard model trained without cut site filters. The predicted occupancy profiles are correlated at 0.98. The rank correlation between the Hinc II model fit on fragments of minimum 2000 bp (comprising 70% of the genome) and its whole-genome counterpart is 0.97. Since the predictions of the long-segment models and the full models are virtually indistinguishable, we use the latter in all further analysis.

**Table 1:** Ranked dinucleotide energies predicted by position-independent models

**Rank**	***In vitro *(Rsa I)**		***In vitro *(Hinc II)**		***In vivo *(Gu & Fire)**		***In vivo *(Valouev et al.)**		***S. cerevisiae in vitro *(Zhang et al.)**		***In vivo (*Embryos)**		***In vivo *(Adults)**		***In vivo *(Germlineless Adults)**	
1	**TT**	1.53	**AT**	1.39	***GC***	1.74	**TT**	1.52	**TT**	1.82	**TA**	1.39	**TA**	1.61	**TA**	1.69
2	**AA**	1.53	**TT**	1.23	***CG***	1.62	**AA**	1.52	**AA**	1.82	**TT**	1.29	**TT**	1.40	**TT**	1.64
3	**AT**	1.45	**AA**	1.23	***GG***	1.51	***CG***	1.48	**TA**	1.14	**AA**	1.29	**AA**	1.40	**AA**	1.64
4	**TA**	1.08	**TA**	0.88	***CC***	1.51	***GC***	1.05	**AT**	1.01	**AT**	1.22	**AT**	1.18	**AT**	1.22
5	GT	0.24	TC	0.36	**TT**	0.4	**TA**	0.68	AG	0.28	CT	0.23	AG	0.23	CT	0.04
6	AC	0.24	GA	0.36	**AA**	0.4	***CC***	0.52	CT	0.28	AG	0.23	CT	0.23	AG	0.04
7	GA	0.11	CA	0.26	**TA**	-0.31	***GG***	0.52	GA	0.2	GA	0.04	TC	-0.09	GA	-0.25
8	TC	0.11	TG	0.26	CT	-0.64	**AT**	0.02	TC	0.2	TC	0.04	GA	-0.09	TC	-0.25
9	TG	-0.06	GT	0.19	AG	-0.64	CT	-0.73	GT	-0.51	TG	-0.04	GT	-0.14	AC	-0.37
10	CA	-0.06	AC	0.19	AC	-0.69	AG	-0.73	AC	-0.51	CA	-0.04	AC	-0.14	GT	-0.37
11	AG	-0.55	AG	-0.08	GT	-0.69	AC	-0.76	TG	-0.56	AC	-0.06	CA	-0.26	TG	-0.55
12	CT	-0.55	CT	-0.08	**AT**	-0.73	GT	-0.76	CA	-0.56	GT	-0.06	TG	-0.26	CA	-0.55
13	***CC***	-0.94	***GG***	-1.33	CA	-0.84	GA	-0.85	***CC***	-0.84	***GG***	-1.08	***CC***	-0.96	***GG***	-0.65
14	***GG***	-0.94	***CC***	-1.33	TG	-0.84	TC	-0.85	***GG***	-0.84	***CC***	-1.08	***GG***	-0.96	***CC***	-0.65
15	***CG***	-1.12	***CG***	-1.58	TC	-0.90	TG	-1.31	***GC***	-1.45	***GC***	-1.59	***GC***	-1.48	***GC***	-1.26
16	***GC***	-2.08	***GC***	-1.95	GA	-0.9	CA	-1.31	***CG***	-1.48	***CG***	-1.80	***CG***	-1.68	***CG***	-1.37

Dinucleotide energies *E*_*w*_ predicted by *N = 2* position-independent models fit on the indicated datasets are shown ranked from highest (least favorable) to lowest (most favorable). The energy of a dinucleotide *w* is defined as $E_{w}=\epsilon_{w_{1}w_{2}}+\epsilon_{w_{1}}+\epsilon_{w_{2}}$, where *w*_*1*_ and *w*_*2*_ are the first and second nucleotides in *w*, and the *ϵ*’s are the fitting parameters of the model (see Methods). Energy contributions are shown in arbitrary units, scaled so that each set of sixteen energies has zero mean and unit variance. A/T dinucleotides, composed only of A and T, are bolded, and G/C dinucleotides, composed only of G and C, are bolded and italicized.

### Read depletion in arms is associated with a simple DNA sequence signature

Remarkably, position-independent models fit to Valouev et al. and Gu & Fire datasets exhibit non-canonical sequence preferences not observed *in vitro* or in other organisms. Specifically, G/C dinucleotides become less favorable than mixed dinucleotides comprised of one A/T nucleotide and one G/C nucleotide; in the case of the Gu & Fire dataset the G/C dinucleotide scores are even more unfavorable than those of the A/T dinucleotides (Table 1). This is consistent with the observation that mixed dinucleotides tend to be enriched at the center and depleted towards the ends of each chromosome (Figure 1C, Additional file 1: Figure S7), making their frequencies anti-correlated with our *in vivo* patterns of nucleosome read coverage. Indeed, *in vivo* but not *in vitro* models reproduce depletion of sequence reads in the autosome arms, whereas the X chromosome occupancy profile is somewhat flatter (Figure 2D,E, Additional file 1: Figure S2, Figure S8 and Figure S9). In contrast, models trained on the three Ercan et al. nucleosome datasets exhibit *in vitro*-like sequence preferences (Table 1, Additional file 1: Figure S6B).

Since our sequence-specific models predict *in vitro* and *in vivo* patterns of read coverage equally well (for example, the *N* = *2* position-independent model predicts Valouev et al. data on which it was trained with r = 0.65, Rsa I model predicts Rsa I data at r = 0.68, and Hinc II model predicts Hinc II data at r = 0.51), read coverage in our *in vivo* experiments is sequence-specific rather than sequence-independent, but non-canonical as compared with *in vitro* studies and other organisms [22,26,27]. We conclude that read depletion on chromosome arms observed in our *in vivo* nucleosome maps may be due to a sequence-specific *in vivo* activity.

### Potential causes of non-canonical *in vivo* nucleosome sequence preferences

According to our experiments, *in vivo* read coverage is lower in chromosome arms, which also have distinct dinucleotide content (Figure 1C, Additional file 1: Figure S7). To exclude the possibility that our fits merely reflect this coincidence, we have refitted the position-independent models only on the central 40% of each chromosome, where the dinucleotide distributions are more uniform. The G/C dinucleotide scores remain unfavorable in our two *in vivo* datasets (Additional file 1: Table S1), implying that the observed de-enrichment of nucleosomal sequence reads in distal regions cannot be caused by effects that occur solely in the chromosome arms.

So, why are loci intrinsically amenable to nucleosome formation depleted of reads *in vivo*? We hypothesize that genome-wide chromatin remodeler activity may explain observed changes in nucleosome occupancy, including read depletion in *C*. *elegans* autosome arms. Indeed, recent work by Moshkin et al. shows that remodelers possess all the necessary characteristics: sequence-specific DNA binding, a tendency to contravene intrinsic nucleosome sequence preferences, and the ability to alter nucleosome occupancy on a global scale [31].

Moshkin et al. studied representatives of four major classes of chromatin remodelers in *D*. *melanogaster*: SWI/SNF, ISWI, CHD/MI2, and INO80. Their work showed that remodelers bind DNA in a sequence-specific manner, and that SWI/SNF, CHD/MI2, and INO80 remodelers push nucleosomes onto low-affinity sites, while ISWI remodelers expel nucleosomes from high-affinity sites. As a result, all four remodelers tend to act against intrinsic nucleosome sequence preferences. Consequently, knocking down remodeler activity significantly improved agreement between nucleosome occupancy observed *in vivo* and occupancy predicted by sequence-specific models trained on *in vitro* data [22,26,52]. Thus, chromatin remodelers can bias apparent nucleosome sequence preferences genome-wide. We infer that remodeling events involving single nucleosomes may contribute to creating large chromatin domains, megabases in size, in *C*. *elegans*.

We have also considered the possibility that changes in sequence preferences are due to localization to the nuclear membrane or direct competition with LEM-2, but this hypothesis is not supported by our data. We compared nucleosome occupancy in LEM-2 “subdomains”, regions identified by Ikegami et al. with considerable LEM-2 occupancy, and “gaps”, where LEM-2 is generally absent [44]. Ikegami et al. argued that chromosomal arms are associated with the nuclear membrane via interactions with subdomain regions, whereas intervening gap regions loop into the nucleus. So, if competition with LEM-2 or attraction to the nuclear membrane were responsible for decreased read coverage in arms, we would expect *in vivo* read coverage to be lower in LEM-2 subdomains than in gaps.

However, we find the opposite trend. Additional file 1: Figure S10 shows histograms of average nucleosome occupancy in gaps, subdomains, and central chromosome regions where LEM-2 is depleted. We observe that *in vivo* read coverage in subdomains is significantly higher than in gaps. In the Gu & Fire data, the gaps have a mean normalized occupancy (Methods) of −0.14 with subdomains at −0.04; from Valouev et al., we find that occupancy in gaps averages to −0.14 while subdomains have a mean occupancy of −0.06. *T*-tests indicate that these differences are not likely to arise by chance (p = 1.3e-04 for Gu & Fire, and p = 5.5e-03 for Valouev et al.). As expected, the large LEM-2 gaps in the centers of each chromosome have high normalized occupancy *in vivo* (0.19 for Gu & Fire and 0.20 for Valouev et al.). On the other hand, Additional file 1: Figure S10 shows that gaps in the chromosomal arms tend to be more nucleosome-covered *in vitro* than subdomains, indicating that DNA sequences in gaps are intrinsically more amenable to nucleosome formation. Thus LEM-2 gaps intrinsically favor nucleosome formation, yet are depleted of reads *in vivo*. These results should not arise if competition with LEM-2 or attachment to the nuclear membrane were responsible for displacing nucleosomes.

### Comparison of bioinformatics and physical models of nucleosome positioning

The global nucleosome coverage bias that makes the fitting parameters in our *in vivo* models deviate from a description of intrinsic histone-DNA sequence preferences affects other types of approaches as well, notably a bioinformatics model similar to the position-independent component of the empirical algorithm developed by Kaplan et al. [26] (Methods). Indeed, nucleosome occupancy profiles predicted with a bioinformatics model trained on *in vitro* data from *C*. *elegans* and yeast are strongly correlated with the corresponding profiles predicted with the *N* = *2* position-independent model (Figure 2B). For Rsa I and Hinc II datasets, shorter restriction fragments and regions around restriction enzyme cut sites were not filtered out as they do not appear to bias the fits. Similarly, a bioinformatics model trained on *in vivo* data from Gu & Fire exhibits strongest correlations with the predictions of the *N* = *2* position-independent models fit to our two *in vivo* datasets. On the other hand, a bioinformatics model trained on the Valouev et al. *in vivo* nucleosome map yields an occupancy profile that is closer to the *in vitro* than *in vivo* predictions of the *N* = *2* position-independent model (Figure 2B). However, the predictive power of the bioinformatics model is lower in this case (r = 0.58 with the Valouev et al. dataset on which it was fit, compared to r = 0.65 for the corresponding *N* = *2* position-independent model). We conclude that regardless of the exact approach used to infer sequence determinants of nucleosome positioning, models of intrinsic nucleosome sequence affinity should not be trained on *in vivo* datasets as their nucleosome distributions may be affected by other factors and the extent of the influence of these factors varies with the details of the computational algorithm.

### The role of 10–11 bp periodic dinucleotide distributions in positioning stable nucleosomes

Nucleosomes that tend to occupy unique genomic positions are found multiple times in our genome-wide nucleosome maps, which combine information from many cells. Once the nucleosomal sequence reads are mapped to the reference genome, their positions become marked by peaks of integer height on the sequence read landscape (Methods). The peak heights thus reflect the degree of unique nucleosome positioning and are likely correlated with nucleosome formation energies, with more stable nucleosomes marked by higher peaks. Therefore, we expect to find sequence signatures of stable nucleosomes in an alignment of all 147 bp-long nucleosomal sequences from the *C*. *elegans* genome that are marked by sequence read peaks with a height above a certain cutoff. We focus on the distribution of dinucleotide frequencies within the nucleosomal site because sequence-specific base stacking energies are thought to assist DNA bending into a nucleosomal superhelix [1,20,53]. The peak height cutoff is an adjustable parameter chosen to select a small subset of the most stable nucleosomes in each dataset (between 0.9% and 1.5% of all sequence reads depending on the experiment, Methods).

**Figure 3:**
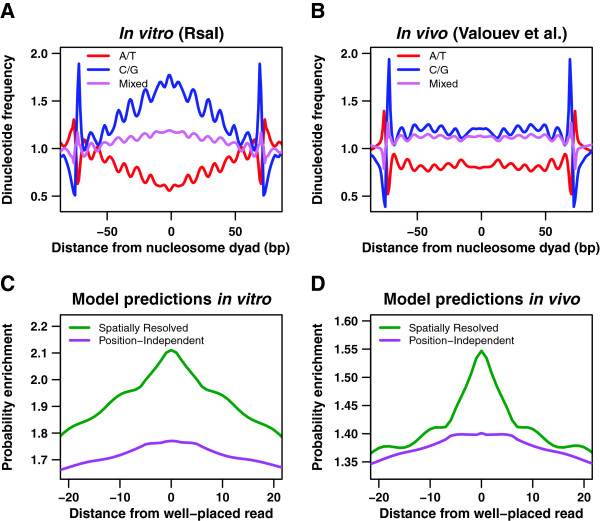
**The role of dinucleotide periodicities in well-positioned nucleosomes. A)** Dinucleotide frequencies in well-positioned *in vitro* nucleosomes. Each curve shows a relative dinucleotide frequency at a given position (with respect to the nucleosome dyad) for the set of well-placed nucleosomes selected from the Rsa I *in vitro* assay (see Methods). Dinucleotide counts used to calculate the frequencies include both forward and reverse strands for each well-placed nucleosome. We define the relative frequency of a group of dinucleotides as the sum of frequencies of all dinucleotides in that group at a given position, divided by the sum of genome-wide frequencies for the same group of dinucleotides. The groups plotted (with a 3-bp moving average) are AA/AT/TA/TT, CC/CG/GC/GG, and mixed dinucleotides (one A or T and one G or C nucleotide). **B)** Dinucleotide frequencies in well-positioned *in vivo* nucleosomes. Same as (A) but using well-placed nucleosomes from the Valouev et al. dataset [39]. **C)** Predicting well-positioned nucleosomes *in vitro*. Each curve shows a probability enrichment predicted by a given model at a given distance from well-placed nucleosomes. Probability enrichment is defined as the predicted probability at a given position, divided by the genome-wide mean of the predicted probability profile. Probability enrichment is averaged over all well-placed nucleosomes in the Rsa I *in vitro* assay; the resulting curves are smoothed with a 7-bp moving average. The two models shown, *N = 2* position-independent (magenta) and spatially resolved (green), were fit on the Rsa I *in vitro* data. **D**) Predicting well-positioned nucleosomes *in vivo*. Same as (C) but with models fit on, and well-placed nucleosomes selected from the Valouev et al. dataset [39].

In Figure 3A and Additional file 1: Figure S11A we plot relative frequencies of dinucleotides at each position within the nucleosomal site. We find that well-positioned *in vitro* nucleosomes are associated with dinucleotide frequency distributions that are very similar to those found in *in vitro* maps from *S*. *cerevisiae*[22,26,27]. Dinucleotide distributions in stable nucleosomes reconstituted *in vitro* on genomic DNA from both organisms are characterized by the overall enrichment of G/C and depletion of A/T dinucleotides in nucleosomal sequences, especially towards the dyad. The dinucleotide frequencies are abruptly reversed in linkers, with sizable jumps across both nucleosome-linker boundaries. Dinucleotide distributions within the nucleosome also exhibit 10–11 bp periodicity of the DNA helical twist [1,20,53]. The similarity of dinucleotide frequency distributions in stable nucleosomes from all *in vitro* experiments is consistent with the fact that the corresponding *N* = *2* position-independent models are also highly similar (Table 1, Figure 2A).

Dinucleotide frequency distributions inferred from *in vivo* well-positioned nucleosomes are significantly different: the difference between G/C and A/T dinucleotide counts is smaller in both nucleosomes and linkers, although the 10–11 bp periodicity inside nucleosomes and the jumps across nucleosome-linker borders are still present (Figure 3B, Additional file 1: Figure S11B). Stable nucleosomes from central regions and chromosomal arms yield virtually identical frequency distributions (data not shown), confirming that their positions are determined locally rather than imposed by global nucleosome coverage trends. In contrast with the *in vitro* situation, where positioning of stable nucleosomes and overall nucleosome occupancy are driven by the same sequence signals, stable *in vivo* nucleosomes do exhibit a slight preference for G/C rather than mixed dinucleotides in the Valouev et al. dataset (Figure 3B). This is in contrast to the dinucleotide ranking generated by the *N* = *2* position-independent model which takes all nucleosomes into account (Table 1). Thus, models trained to reproduce *in vivo* nucleosome coverage profiles in *C*. *elegans* have to balance the need to capture the locations of stable nucleosomes with the need to account for the relative depletion of sequence reads from chromosomal arms and G/C-rich sequences in general.

We have previously shown that it is not necessary to model 10–11 bp dinucleotide frequency periodicities in *S*. *cerevisiae* if the goal is to reproduce nucleosome occupancy profiles: Simple position-independent models which capture the differences between average dinucleotide frequencies in nucleosomes and linkers are sufficient and have relatively few fitting parameters (13 in the *N* = *2* position-independent model vs. 1684 in a spatially resolved model which assigns distinct mono- and dinucleotide energies at each position within the nucleosomal site) [22]. Indeed, yeast occupancy profiles predicted using *N* = *2* position-independent and spatially resolved models are highly correlated (r = 0.98 genome-wide). This is also true in *C*. *elegans*, where both types of models yield virtually identical occupancy profiles (Figure 2C).

However, spatially resolved models are better at predicting positions of stable nucleosomes with single-bp precision, both *in vitro* and *in vivo*, as seen in Figure 3C,D and Additional file 1: Figure S11C,D, which show predicted probabilities to start a nucleosome in the vicinity of well-placed nucleosomes marked by higher sequence read peaks (Methods). Thus, the affinity of the histone octamer for genomic DNA is largely controlled by its dinucleotide content, which is relatively easy to modulate even if DNA sequence is constrained by the need to encode functional elements such as exons or transcription factor binding sites. The affinity may be further increased and the nucleosome position refined through 10–11 bp periodic dinucleotide distributions that either exist in the genome for other reasons or have specifically evolved to place nucleosomes more precisely. The former idea is supported by the observation of prominent 10–11 bp dinucleotide periodicities in sequences of well-positioned nucleosomes assembled *in vitro* on the *E*. *coli* genome, which has not evolved for nucleosome formation [22].

We find noticeable 10–11 bp dinucleotide periodicities in well-positioned nucleosomes from all yeast, *E*. *coli* and *C*. *elegans* large-scale maps, regardless of the extent of the bias in the overall dinucleotide content (Figure 3A,B, Additional file 1: Figure S11A,B) [22]. However, both types of signals are present in stable nucleosomes obtained by *in vitro* reconstitution, which presumably occupy the lowest free energy positions available genome-wide (alternatively, increased G/C content in nucleosomes assembled *in vitro* may be an experimental artifact [28]). Thus, our analysis suggests that both signals may increase binding affinity, and that 10–11 bp periodic sequences are ubiquitous enough in the genome to be readily utilized by the subset of stable nucleosomes. Direct measurements of free energies of nucleosome formation are necessary in order to compare the magnitudes of energetic contributions associated with each type of sequence signal.

### Stable nucleosomes do not occupy unique positions *in vivo*

*In vitro* selection experiments for histone-DNA binding affinity, followed by direct measurements of free energies of nucleosome formation, indicate that nucleosome free energies fall within a range of several kcal/mol [13-15]. Energies of this magnitude are readily available in the cell, e.g. through ATP hydrolysis utilized by chromatin remodeling enzymes to reposition or unfold nucleosomes [54]. Although bulk *C*. *elegans* nucleosomes may be repositioned *in vivo*, or initially deposited differently, there could be a core of stable nucleosomes which occupy unique genomic locations under all experimental conditions. These positions would be dictated purely by DNA sequence rather than external factors such as the concentration of chromatin remodelers in the nucleus, and the stable core would thus be observed both *in vitro* and *in vivo*. Figure 3A,B and Additional file 1: Figure S11A,B already indicate that positions of stable nucleosomes are not strongly conserved – if that were the case, distinct *in vitro* patterns of dinucleotide frequencies in stable nucleosomes would be more closely reproduced *in vivo*.

**Figure 4:**
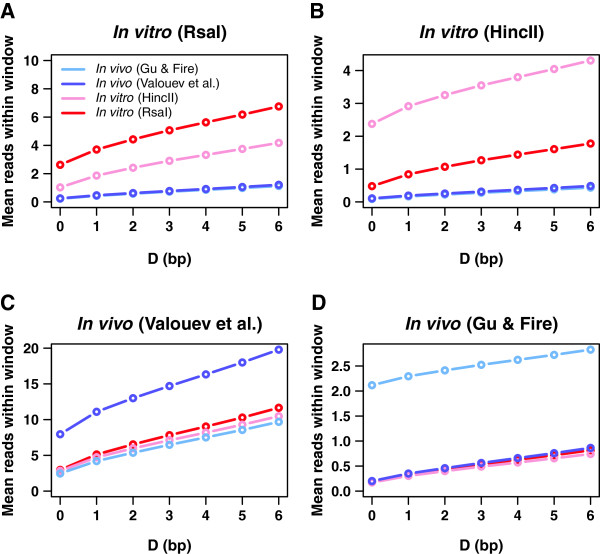
**Overlap between well-positioned nucleosomes in different datasets. A)** Each curve shows the average number of reads observed in the indicated dataset within *D* bp of all well-placed nucleosomes from the Rsa I *in vitro* assay. **B)** Same as (A), but with respect to well-placed nucleosomes in the Hinc II *in vitro* assay. **C)** Same as (A), but with respect to well-placed nucleosomes in Valouev et al. *in vivo* dataset [39]. **D)** Same as (A), but with respect to well-placed nucleosomes in Gu & Fire *in vivo* dataset [38]. Note that in each panel, the vertical scale of each curve is normalized to match the total read-coverage of the dataset from which the well-placed nucleosomes are drawn. Sequence read cutoffs and filtering procedures used to define well-positioned nucleosomes are described in Methods.

We sought to address this question more directly by computing how many nucleosomes from dataset *B* occur, on average, within *D* bp of stable nucleosomes in dataset *A* (Figure 4). The windows of 2*D* + 1 bp width centered on stable “*A*” nucleosomes are used to account for MNase digestion artifacts [55], which preclude identification of nucleosome locations with single-bp accuracy. We find that stable nucleosomes from the Rsa I *in vitro* assay are partially reproduced in the Hinc II assay, and *vice versa*. Indeed, three-base windows (*D* = 1 bp) centered on well-positioned Rsa I nucleosomes (themselves marked by 5.04 sequence reads on average, as shown in the *D* = 0 data point in the red curve from Figure 4A) contain 4.08 Hinc II reads on average, 81% of the total. Note that Hinc II sequence read coverage was rescaled to equal that of Rsa I. Likewise, on average 37% of well-positioned Hinc II nucleosomes are found within three-bp windows in the Rsa I dataset (Figure 4B). All regions in the vicinity of Rsa I and Hinc II cut sites were excluded from this analysis (Methods). The corresponding *in vivo* fractions are approximately seven times smaller, suggesting that the majority of *in vitro* stable nucleosome positions are not occupied *in vivo*. In addition, our two *in vivo* datasets are not closest to each other (Figure 4C,D). Overall, many well-positioned *in vivo* nucleosomes are distant from intrinsically favorable loci, and their locations appear to vary from one experiment to another. Furthermore, since reproducibility of stable nucleosome positions in the two *in vitro* experiments was not perfect, it is possible that thermodynamic equilibrium was not fully reached on experimental timescales.

### Chromatin organization in transcribed regions

We examined the effect of transcription on nucleosome occupancy by evaluating the performance of our models in genic regions. If transcriptional activity leads to nucleosome rearrangements and displacements, nucleosome occupancy in genes should adhere more weakly to intrinsic nucleosome sequence preferences. However, the power of our *in vitro* models to predict *in vivo* occupancy in genes compares well with their genome-wide predictive power: the Rsa I model predicts Valouev et al. read coverage at r = 0.50 in genes and r = 0.48 across the genome. The Hinc II model predicts Valouev et al. read coverage at r = 0.38 in genes and r = 0.37 across the genome. Comparisons using Gu & Fire data yield similar results (data not shown). Thus our ability to predict nucleosome occupancy is approximately the same in genic regions and genome-wide.

**Figure 5:**
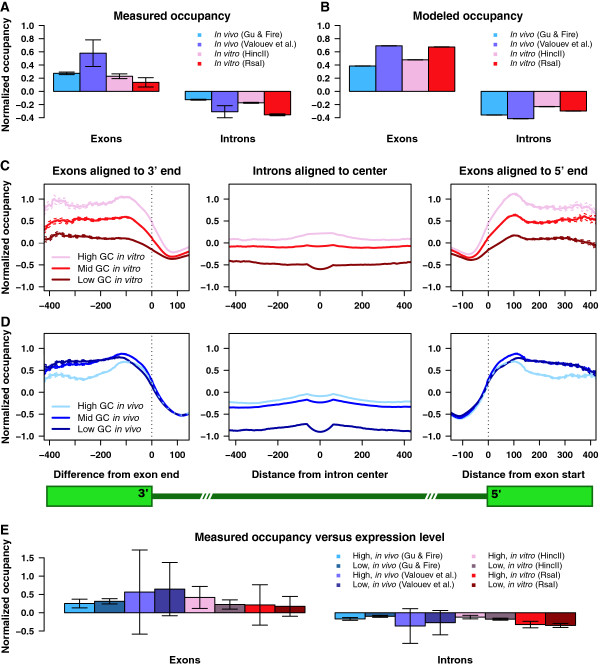
**Nucleosome organization in exons and introns. A)** Barplot of exon and intron nucleosome occupancies. The mean normalized nucleosome occupancy in introns and exons for each dataset is plotted. Error bars show the standard error. **B)** Barplot of predicted exon and intron nucleosome occupancies. Same as (A), but using normalized nucleosome occupancy profiles predicted by *N = 2* position-independent models fit on the indicated datasets. **C)***In vitro* nucleosome occupancy in exons and introns grouped by GC content. Exons and introns were divided into three equally sized groups of high, medium, and low GC content. Introns were aligned on their center, and exons were aligned to their 3’ ends (left) and 5’ ends (right). Mean normalized nucleosome occupancy in each group is plotted against the distance from the center for introns, and the distance from either the 3’ or the 5’ boundary for exons. Averages *x* bases upstream of the 3’ boundary or downstream of the 5’ boundary are calculated only among exons of length ≥ *x*. The average intron nucleosome occupancy a distance *x* from the intron center is calculated only among introns of length ≥ *2x*. Dashed curves show standard errors of the mean. The nucleosome occupancy profile is from the Rsa I *in vitro* assay. **D)***In vivo* nucleosome occupancy in exons and introns grouped by GC content. Same as (C), but using *in vivo* data from Valouev et al. [39]. **E)** Exon and intron nucleosome occupancy grouped by gene expression levels. Same as (A), except that exons and introns are from the genes with high and low expression levels. Expression levels were inferred from SAGE data. All tagged genes were ranked by the abundance of SAGE tags, with high and low expression groups corresponding to the top and bottom 10%, respectively (see Methods).

Previous studies have found a correlation between nucleosome occupancy patterns and exon-intron organization, implicating chromatin in exon recognition during co-transcriptional splicing [36,37]. Consistent with these studies, we find that nucleosome occupancy is above average in exons and below average in introns, both *in vitro* and *in vivo* (Figure 5A). These trends are also captured by the *N* = *2* position-independent models (Figure 5B), showing that they can be partially ascribed to the systematic differences between exon and intron dinucleotide content. The success of the *in vitro* models in reproducing this pattern suggests that exons and introns contain sequence signals that, respectively, intrinsically favor and disfavor nucleosome formation. As shown in Additional file 1: Figure S12, the average distribution of sequence reads around exon-intron boundaries shows a pronounced peak, which corresponds to numerous well-positioned nucleosomes extending from the exon-intron boundary into the exon, both *in vivo* and *in vitro*. Since these nucleosomes demarcate the exon boundaries with high precision, it is conceivable that they play a role in directing transcriptional and splicing machinery. Interestingly, there are also small secondary peaks which correspond to the nucleosomes that start at the boundary but extend into introns rather than exons. Note that the restriction enzyme cut site filters are not applied to *in vitro* datasets in this section since focusing on long restriction fragments leaves our conclusions unaffected (data not shown), yet lowers their statistical significance because a sizable fraction of transcribed regions is removed from the analysis.

When exons and introns are divided into three groups of equal size by G/C content, the *in vitro* occupancy follows the expected pattern in which nucleosomes preferentially assemble on G/C-rich sequences in both introns and exons (Figure 5C, Additional file 1: Figure S13A). However, these intrinsic preferences are substantially modified *in vivo* (Figure 5D, Additional file 1: Figure S13B), as previously noted [36,37]. In both *in vivo* experiments, high-G/C exons tend to be nucleosome-depleted, while the difference in occupancy between medium-G/C and low-G/C exons is either eliminated (Figure 5D) or reversed (Additional file 1: Figure S13B). In contrast, *in vitro* and *in vivo* intron occupancy patterns are relatively close to each other.

Next we inquired whether the *in vivo* redistribution of nucleosomes covering exon and intron sequences is related to the observed nucleosome de-enrichment in chromosomal arms. We have repeated the above G/C analysis for the genes located in the central 40% of each chromosome, where the global nucleosome coverage profiles are essentially flat (Additional file 1: Figure S14). As expected, the correlation between nucleosome occupancy and G/C content is still observed *in vitro*, while *in vivo* there is again nucleosome depletion from exons with high G/C content. We conclude that de-enrichment of read coverage in chromosomal arms is not correlated with the observed *in vivo* changes in nucleosome occupancy over exons.

If nucleosomes in exons are actively manipulated by the components of transcriptional and splicing machinery, one could expect the corresponding nucleosome occupancy to depend on the expression state of the gene. However, we do not find highly significant differences between nucleosome occupancies of exons and introns in strongly and weakly expressed genes, either *in vitro* or *in vivo* (Figure 5E). Gene expression levels were quantified as SAGE mRNA transcript counts [56,57] (Methods). This observation is in contrast with a previous finding in activated human T cells, where a negative correlation between nucleosome occupancy and transcript expression levels was reported both *in vivo* and by using nucleosome occupancy predictions based solely on DNA sequence as a proxy for the *in vitro* experiment [36]. Lower nucleosome occupancy was also found in transcribed vs. non-transcribed genes in resting T cells [37]. Weak correlation between nucleosome occupancy and gene expression levels is, however, supported by our earlier observations in yeast, where most changes in gene expression were not accompanied by nucleosome remodeling in promoters and coding regions [58,59].

If nucleosomes participate in the process of co-transcriptional splicing, one might expect a correlation between the strength of 5’ and 3’ splice sites and the degree of nucleosome coverage. We have scored each splice site using a position-specific weight matrix (PWM) based on the alignment of all 5’ and 3’ splice sites in the *C*. *elegans* genome (Methods). We find that the difference between *in vitro* nucleosome occupancies of exons with top 10% and bottom 10% of PWM-scored 5’ splice sites (located at exon 3’ ends) is rather small (Additional file 1: Figure S15A). For top 10% and bottom 10% of PWM-scored 3’ splice sites (located at exon 5’ ends), the difference is more pronounced: exons with weak splice sites tend to be more nucleosome-covered *in vitro*. The differences between weak and strong splice sites become more noticeable *in vivo*, with strong 3’ splice sites correlated with nucleosome depletion and strong 5’ splice sites correlated with nucleosome enrichment relative to the corresponding weak sites (Additional file 1: Figure S15B). Thus splicing factors may interact with nucleosomes in a way that promotes nucleosome occupancy over strong donor sites and depletes nucleosomes from strong acceptor sites.

Previous studies have shown that nucleosome occupancy in resting and activated T cells on average increases toward the 3’ end of genes [36], possibly reflecting partial transcription events that might be accompanied by stronger nucleosome depletion from 5’ gene ends. Additional file 1: Figure S16, which shows average nucleosome occupancy in exons plotted by exon number, exhibits a similar trend, although its statistical significance is rather low. This trend does not appear to be encoded through intrinsic histone-DNA interactions as it is not observed *in vitro*. However, it is captured by the *N* = *2* position-independent models trained on *in vivo* data and thus capable of reproducing chromosome-wide patterns of nucleosome read coverage. This observation suggests that the slightly higher occupancy of downstream exons may be in part due to the fact that longer, multi-exon genes occur more frequently in central regions. Indeed, genes located in the central 40% of *C*. *elegans* chromosomes have 6.29 ± 0.04 exons on average, whereas genes located in the 20% terminal domains have only 5.69 ± 0.04 exons. However, we cannot rule out a possibility that the relative depletion of nucleosomes from 5’ exons is due to such exons being involved more frequently in transcriptional events.

## Conclusions

We have analyzed sequence determinants of nucleosome positioning and occupancy using two large-scale maps of *in vitro* nucleosomes reconstituted on genomic DNA from the nematode worm *C*. *elegans*, a complex metazoan organism. Intrinsic histone-DNA sequence specificity explored in these experiments was first studied using biophysical models in which the effective free energy of nucleosome formation depends simply on the total number of mono- and dinucleotides in nucleosomal sequences and not on their position with respect to the nucleosomal dyad (*N* = *2* position-independent models, see Methods) [22,35,51]. We have found that nucleosome occupancy predictions of these models are highly correlated with the predictions of the same model trained on a high-throughput map of nucleosomes assembled *in vitro* on genomic DNA from a single-cell eukaryote *S*. *cerevisiae* (Figure 2A) [27]. Thus, reassuringly, our approach yields a consistent picture of sequence signals responsible for nucleosome occupancy in the absence of confounding factors such as chromatin remodeling enzymes. In this picture, nucleosomes prefer to occupy G/C-enriched and A/T-depleted sites, while in linkers the preferences are reversed.

Next we have asked whether these intrinsic nucleosome positioning rules are respected in chromatin extracted from live *C*. *elegans* cells [38,39]. Surprisingly, G/C dinucleotides, which favor nucleosome formation the most *in vitro*, become much less favorable *in vivo* according to our experiments but not according to those from Ercan et al. [40] which result in *in vitro*-like occupancy profiles (Table 1, Additional file 1: Figure S6). This discrepancy can be attributed to substantial differences between our experimental protocols. The deviation from *in vitro* behavior has not been observed in yeast [22,26] and thus may constitute a unique signature of *C*. *elegans* chromatin. Significant disruptions of intrinsic nucleosome sequence preferences in *C*. *elegans in vivo* have been previously reported [22,26]. For example, in comparing nucleosome preferences for DNA 5-mers, Kaplan et al. found that both CCGGC and CGGCA are favorable in yeast *in vitro* but strongly unfavorable in *C*. *elegans in vivo*, affirming our conclusion that G/C dinucleotides in general are less favorable in *C*. *elegans*. Our *in vitro* assays rule out the possibility that these changes are due to unusual mechanical properties of the *C*. *elegans* genome [50].

The *in vivo* changes in apparent sequence specificity are associated with a global pattern of nucleosome read coverage in *C*. *elegans*: coverage in chromosomal arms is lower than in central regions (Figure 1A, Additional file 1: Figure S2 and Figure S3). This large-scale arrangement is not observed *in vitro* (Figure 1B, Additional file 1: Figure S2 and Figure S4), and is reminiscent of the broad patterns of histone modifications and meiotic recombination rates in the *C*. *elegans* genome [47]. The observed change in apparent sequence affinity *in vivo*, in conjunction with broad patterns of dinucleotide content in the *C*. *elegans* genome, appears to create these chromatin domains; if read depletion in the arms caused the observed changes, we would expect *in vivo* models trained only on chromosome centers to exhibit more canonical sequence preferences, but such is not the case. Rather, our analysis supports the hypothesis that chromatin remodelers direct nucleosomes away from G/C-rich DNA genome-wide, creating chromosome-scale occupancy patterns. As a result, both DNA sequence preferences and *in vivo* remodeling activities contribute to the final nucleosome disposition in living cells.

Alternatively, differential recovery of sequence reads in the arms vs. central regions could be caused by distinct patterns of post-translational modifications, as well as consistent differences in chromatin tertiary structure and histone variant usage. These factors could affect MNase-mediated recovery of mononucleosome fragments, potentially amplifying or masking nucleosome occupancy differences. We note that although read depletion in chromosome arms may be partially due to such effects, the genome-wide distribution of mapped reads in our *in vivo* experiments is globally sequence specific in a way not observed *in vitro* or in other organisms, and thus may reflect an essential feature of *C*. *elegans* chromatin.

Although *N* = *2* position-independent models capture global nucleosome occupancy trends, they disregard more subtle sequence signals such as 10–11 bp periodic dinucleotide distributions which are thought to facilitate bending of nucleosomal DNA into the superhelical shape [1,53]. We find that including such signals into the models helps predict well-positioned nucleosomes (Figure 3C,D, Additional file 1: Figure S11C,D), albeit at the price of many more fitting parameters. Thus, while more detailed spatially resolved models predict occupancy profiles that are nearly identical to their position-independent counterparts, they offer sizable improvement in predicting positions of stable nucleosomes with base-pair precision. Interestingly, well-positioned *in vivo* nucleosomes tend to occupy sequences that are slightly enriched in G/C dinucleotides (Figure 3B, Additional file 1: Figure S11B). Focusing on whether the locations of well-positioned nucleosomes are similar in all datasets, we find that *in vitro* nucleosomes marked by higher sequence read peaks have relatively few *in vivo* counterparts, even if imprecision of MNase digestion is taken into account (Figure 4A,B). Thus it appears that the majority of well-positioned *in vivo* nucleosomes do not occupy thermodynamically favorable sites observed *in vitro* (assuming that *in vitro* nucleosomes themselves have equilibrated at physiologicl salt concentrations [28]) – rather, they are found at locally optimal sites. These sets of locally optimal positions differ between our two *in vivo* experiments, calling into question the idea that *in vivo* rather than *in vitro* nucleosome positions are in fact in thermodynamic equilibrium. Our observations are supported by a recent study in which artificial chromosomes with foreign genomic DNA were transformed into *S*.*cerevisiae*[60]. Subsequent nucleosome mapping revealed numerous disagreements between nucleosome occupancy profiles in native and foreign *in vivo* contexts.

Finally, we have studied chromatin structure in the vicinity of exons and introns. Previous studies have found a link between nucleosome positioning and exon-intron architecture based on data from human cells, reporting widespread nucleosome depletion from introns and enrichment in exons which did not correlate with intrinsic preferences for G/C-rich sequences [36,37]. We confirm these findings in *C*. *elegans* (Figure 5D, Additional file 1: Figure S13B) and further discover that the G/C preferences are restored *in vitro* (Figure 5C, Additional file 1: Figure S13A). However, we do not see a strong correlation with either gene expression levels or splice site strength (Figure 5E, Additional file 1: Figure S15), suggesting that the direct action of transcriptional and splicing machinery is not a major contributor to the exon-intron chromatin architecture. Rather, we surmise that the same mechanism is responsible for both large-scale sequence read depletion from chromosomal arms and small-scale sequence read depletion from exons with high G/C content.

In summary, we have compared *in vitro* and *in vivo* nucleosome maps in *C*. *elegans* to discover striking functional differences on multiple scales. As the high-coverage mononucleosome sequencing data on higher organisms accumulates, we look forward to learning whether the observed *in vivo* rearrangements are generic in complex metazoans, or whether *C*. *elegans* stands apart in its extent of chromatin remodeling and its degree of changes in global chromatin architecture.

## Methods

### Reconstitution of *in vitro* nucleosomes (invitrosomes)

Naked genomic DNA from wild-type *C*. *elegans* (N2 strain) was isolated by digesting flash-frozen worms with proteinase K (Roche, 2mg/ml final concentration) in worm lysis buffer (0.1M Tris–HCl at pH 8.5, 0.1 M NaCl, 50 mM EDTA, 1% SDS) at 65°C for 45 min followed by phenol, phenol/chloroform, chloroform extraction and ethanol precipitation. RNA was removed by digesting the isolated nucleic acid with RNAse A (Roche) followed by phenol/chloroform, chloroform extraction and ethanol precipitation. To produce DNA templates for both the Rsa I and Hinc II experiments, 40 μg of high-molecular weight genomic DNA was digested with 200 units of either restriction enzyme Rsa I or Hinc II (New England BioLabs) with the supplied buffers and 1X BSA (New England BioLabs). Digestion proceeded at 37°C for two hours followed by phenol, phenol/chloroform, chloroform extraction and ethanol precipitation, resulting in complete digestion as assayed on a 1% UltraPure Agarose (Invitrogen) gel (Additional file 1: Figure S17). A continuous smear of fragments was seen for both digestions with a distribution of fragments lengths visually estimated to be centered upon and enriched around ~850 bp and ~3500 bp for the Rsa I and Hinc II digestions, respectively.

The Rsa I and Hinc II DNA digestions were assembled with recombinant *Xenopus* histones (a gift from Geeta Narlikar) into nucleosomes as described previously [61] at a 1.1:1 molar ratio of DNA to histone octamer, such that on average one nucleosome would form per 850 bp or 3500 bp of DNA for the Rsa I and Hinc II reconstitutions, respectively. Specifically, 9.13 μg of DNA and 1.45 μg of histone octamer (for Rsa I) and 22.00 μg of DNA and 0.88 μg of histone octamer (for Hinc II) were reconstituted in a total volume of 200 μl. We call these *in vitro* reconstituted nucleosomes invitrosomes and will refer to them as such hereafter.

### Isolation of invitrosome core DNA fragments

Invitrosome core DNAs from both Rsa I and Hinc II reconstitutions were isolated by diluting 60 μl of the respective invitrosomes into a total volume of 200 μl containing 5 mM MgCl2, 5 mM CaCl2, 70 mM KCl and 10 mM Hepes at pH 7.9 (final concentrations) and digesting with 20 units of MNase (Roche) resuspended at 1 U/μl for 15 min at room temperature. The digestion was stopped by adding an equal volume of 3% SDS, 100 mM EDTA and 50 mM Tris. Histones were removed by treating with one-tenth volume proteinase K (20 mg/ml in TE at pH 7.4) for 30 min at 50°C followed by phenol/chloroform and chloroform extractions and ethanol precipitation. In order to obtain enough sample for the Hinc II reconstitution, this procedure was repeated three times to process the entire Hinc II invitrosome sample and then pooled together in a total volume of 30 μl (the single processing of the Rsa I sample was also in a total volume of 30 μl). Invitrosome DNA cores were assayed for complete digestion and isolated on a 2% UltraPure Agarose (Invitrogen) gel run at 100 V for 1 h, followed by DNA extraction from the gel using a QIAquick Gel Extraction Kit (Qiagen) and following the standard protocol, with the exception of allowing the isolated gel sample to incubate in Buffer QG at room temperature until dissolved, rather than heating the sample at 50°C as recommended by the manufacturer.

### Illumina library preparation and sequencing

The Rsa I and Hinc II libraries were prepared by processing the invitrosome core DNA fragments using Illumina Genomic DNA Sample Prep Kit (Illumina 2007 Rev. A). Fragment end repair, adapter ligation and library amplification were all done according to the kit instructions “Preparing Samples for Sequencing Genomic DNA” (Illumina 2007 Rev. A), with exceptions to the protocol mentioned below. Since our libraries are composed of ~147 bp DNA cores rather than intact genomic DNA, the protocol was started at the “Perform End Repair” step (page 11). At this step, 20 μl (~200 ng) of the Rsa I invitrosome core DNA sample and 30 μl (~50 ng) of the Hinc II invitrosome core DNA sample were used. For the Rsa I sample, 10 μl of extra water was added to achieve the 30 μl volume prescribed by the protocol. At the “Ligate Adapters to DNA Fragments” step the purification was performed with the QIAquick PCR Purification Kit (Qiagen) rather than MinElute PCR Purification Kit, and eluted in 30 μl of EB rather than 10 μl, with 10 μl of the sample used in the next step. Additionally, a no-DNA control sample was processed in parallel to the Rsa I and Hinc II samples. After library preparation, each library was sequenced using a single lane of the Illumina GAII sequencer, resulting in 9.5 million and 5.5 million raw 36-bp reads for the Rsa I and Hinc II libraries, respectively.

### *In vitro* and *in vivo* nucleosome positioning maps

The *in vitro* reads were mapped to the WS190 *C*. *elegans* genome using Bowtie (http://bowtie-bio.sourceforge.net) [62], aligning the first 25 bases out of 36 and allowing for up to one mismatch if the perfect match could not be found. With Ercan et al. reads [40], we aligned the 36 bases of each read allowing up to one mismatch in each half of the pair, again using Bowtie. We required a minimum of 130 bases and a maximum of 200 bases between paired reads. In cases where these procedures yielded *M* equivalent locations, we assigned reads of height *1*/*M* to each location. Mapped reads *in vivo* are as reported in Gu and Fire [38] and Valouev et al. [39]. The read coverage for each dataset is 0.086 reads per base in Rsa I, 0.048 reads per base in Hinc II, 0.040 reads per base in Gu and Fire, 0.44 reads per base in Valouev et al., 0.58 reads per base in embryos, 0.84 reads per base in adults, 0.67 reads per base in germlineless adults, and 0.58 reads per base in the nucleosome-free control experiment. The last four datasets are from Ercan et al. [40].

### Pre-processing of nucleosome sequence reads

We extend all mapped reads to the 147 bp canonical nucleosome length and combine reads from both strands as previously described [22]. For sequence reads mapped onto the forward (5’) strand, we interpret the first base of each read as the start position of the 147 bp nucleosomal site. For sequence reads mapped onto the reverse (3’) strand, we interpret the last base of the read as the end position of a 147 bp nucleosome. For paired-end reads from Ercan et al. [40] (including the nucleosome-free MNase digestion control), we assign a nucleosome dyad to the genomic coordinate halfway between each pair’s start and end coordinates, and extend the nucleosome 73 bases in either direction. This procedure yields the number of nucleosomes that start at each genomic bp (the sequence read profile), as well as the number of nucleosomes that cover a given bp (the nucleosome coverage profile).

We control for sequencing and mapping artifacts by filtering out regions with anomalously high and low nucleosome coverage. We occasionally observe large gaps in sequence read profiles, possibly due to repetitive regions in the genome to which reads cannot be mapped uniquely, or to sequencing artifacts. We consider any stretch of ≥ 1000 bp without mapped reads to be anomalous and exclude such regions from further analysis. We also find regions where the read coverage is uncharacteristically high. We exclude such regions according to the following algorithm: For each chromosome, we find the average number of reads per bp. Next, for each bp we calculate the running average number of reads in a window extending 75 bp in each direction. If this running average is more than three times the chromosome-wide mean, we flag the region which extends out from the identified point in both directions until the running average equals the mean, and remove this region from consideration. Each excluded region is extended 146 bp upstream so that there is no contribution to the nucleosome energy from filtered regions. Finally, we create a filter which marks the union of all excluded regions.

To control for the end effects caused by nucleosome assembly on short DNA fragments, an additional filter was applied to *in vitro* data, excluding 200 bp on either side of each restriction enzyme cut site used in the respective assay. The filter was applied to all cut sites, which on average occur once per 490 bp for Rsa I (GTAC) and once per 2109 bp for Hinc II (GTYRAC). These lengths are shorter than those found experimentally (see above) because not every site has been cut. If applied, the filter removes 87.7% of genomic bps from the Rsa I dataset and 19.0% of genomic bps from the Hinc II dataset. Since the number of bps removed is considerable enough to affect statistical significance of our findings, the filter was applied only if the results changed substantially (Figure 1D, Additional file 1: Figure S6A, Figure 3, Figure 4).

Since Valouev et al. mapped sequence reads to the WS170 version of the *C*. *elegans* genome [39], they had to be remapped to the WS190 version. We used BLASTZ [63] to compare chromosomes from both genomes, identifying homologous regions and locations where bps should be inserted or deleted to transform the WS170 genome into WS190. Where the alignment indicated an insertion, we inserted a segment of appropriate length with zero reads into the WS170 read profiles. Likewise, for deletions, we removed bases from the original read profiles. The altered read profiles were then run through the standard filtering procedure with one difference: locations within 147 bases of insertions or deletions were also filtered out.

We identified well-positioned nucleosomes in each sequence read profile using the following cutoffs: Rsa I, nucleosomes marked by reads with height 4 and above (1.3% of all reads); Hinc II, nucleosomes marked by reads with height 3 and above (1.1% of all reads); Gu & Fire [38], nucleosomes marked by reads with height 3 and above (0.9% of all reads); Valouev et al. [39], nucleosomes marked by reads with height 9 and above (1.5% of all reads). To minimize end effects, all sequence reads within 200 bp of every restriction enzyme cut site were excluded from *in vitro* datasets, as described above.

We smoothed the sequence read and nucleosome coverage profiles by replacing the number of nucleosomes starting at each bp with a Gaussian centered on that bp. The area of the Gaussian is equal to the number of sequence reads starting at that position, and its σ is set to either 2 or 20 depending on subsequent modeling, as described below. The smoothed sequence read profile is constructed as a superposition of all such Gaussians. Gaussian smoothing is necessary because current levels of sequence read coverage lead to large deviations in the number of nucleosomes located at neighboring bps, contrary to the expectation that such nucleosomes have very similar binding affinities because they occupy nearly identical sites. In addition, the smoothing procedure reflects a lack of bp precision in MNase digestion assays, which results in the uncertainty of the interpretation of sequence read coordinates as nucleosome start or end positions. Finally, we normalize sequence read and nucleosome coverage profiles by the highest value of the nucleosome coverage on each chromosome (excluding the filtered regions). We interpret the resulting normalized profiles as the probability to start a nucleosome at a given bp (the nucleosome probability profile) and the probability that a given bp is covered by any nucleosome (the nucleosome occupancy profile).

### Prediction of nucleosome energetics from high-throughput sequencing maps

We derive nucleosome formation energies directly from the Gaussian-smoothed probability and occupancy profiles. Our model rigorously treats intrinsic histone-DNA interactions and steric exclusion [22]:

(1) $$\begin{array}{l} \frac{E_{i}-\mu }{k_{B}T}=\log\frac{1-O_{i}+P_{i}}{P_{i}}\\ \;+\sum_{j=i}^{i=146}\log\frac{1-O_{j}}{1-O_{j}+P_{j}}\;,\;i=1,\ldots ,L-146 \end{array}$$

Here *E*_*i*_ is the nucleosome energy at bp *i, μ* is the chemical potential of histone octamers, *k*_*B*_*T* is the product of the Boltzmann constant and room temperature, *L* is the number of bps in the DNA segment, *P*_*i*_ is the probability to start a nucleosome at bp *i* , and *O*_*i*_ is the nucleosome occupancy of bp *i*$\left(O_{i}=\sum_{j=i-146}^{i}P_{j}\right)$.

We establish the degree of correlation between nucleosome energies and sequence features found in nucleosomal and linker DNA by fitting the energies to one of the two sequence-specific models. The position-independent model of order *N* is given by [22]:

(2) $$\frac{E_{i}-\mu }{k_{B}T}=\epsilon^{0}+\sum_{n=1}^{N}\sum_{\left\{a_{1\ldots }a_{n}\right\}}^{3^{n}}m_{a_{1}\ldots a_{n}\;}^{i}\epsilon_{a_{1}\ldots a_{n}}+r_{i}$$

Here *N* is the maximum word length, *ϵ*^*0*^ is a sequence-independent offset, $m_{a_{1\ldots }a_{n}}^{i}$ is the number of times a word of length n with sequence *a*_*1*_…*a*_*n*_ was found within the nucleosome that started at bp *i*, *ϵ*_*a1*…__*an*_ are word energies, and *r*_*i*_ is the residual. The word energies are constrained by $\sum_{a_{i}}\epsilon_{a_{1}\ldots a_{n}}=0,\forall i=1\ldots n$, which leaves *3*^*n*^ independent words of length *n*. We exclude all words that extend into 3 terminal bps on each end of the 147 bp-long nucleosomal site from our counts. We use only the *N* = *2* position-independent model in this work.

The spatially resolved model is defined by [22]:

(3) $$\frac{E_{i}-\mu }{k_{B}T}=\epsilon^{0}+\sum_{j=i+3}^{i+143}\epsilon_{a_{j}a_{j+1}}^{j}+\sum_{j=i+3}^{i+144}\epsilon_{a_{j}}^{j}+r_{i},$$

where the mono- and dinucleotide energies are constrained as above, but separately for each position within the nucleosomal site. We use Gaussian smoothing with *σ* = 20 for position-independent models and *σ* = 2 for spatially resolved models.

Eqs. (2) and (3) define linear models which we fit against nucleosome energies using the lm function from R statistical software (http://www.r-project.org). For computational reasons, the genome is divided into several segments of equal size and a separate model is trained on each segment. The final energy of each word is the average over all models fitted on separate segments. We divide the genome into 6–8 segments for position-independent models, depending on the percentage of bps filtered out in a given dataset. For spatially resolved models, the computational effort is too large to fit all models on the entire genome. Instead, we fit only Gu & Fire *in vivo* data genome-wide, dividing the nucleosome occupancy profile into 405 segments of equal size and performing a cross-correlation study which shows that predicted energies averaged over >40 randomly picked segments no longer change appreciably (Additional file 1: Figure S18). Therefore the other spatially resolved models were fit on 40 segments randomly chosen out of 405, which comprise approximately 10% of the *C*. *elegans* genome. We restore the dynamic range of fitted energies by rescaling their variance to the variance of the nucleosome energies on which they were trained, separately for each chromosome. Finally, we predict nucleosome probabilities and occupancies from fitted energies using a standard recursive algorithm [22,53].

### Normalized occupancy

To enable unbiased comparisons between different experiments and predictions, we sometimes further rescale nucleosome occupancy profiles. We find the mean *μ* and variance σ^2^ of each profile in unfiltered regions and scale that profile so that the mean becomes zero and the variance becomes one: *N*_*i*_ = (O_*i*_ − *μ*)/*σ*, where *N*_*i*_ is the normalized occupancy at position *i* and *O*_*i*_ is the un-normalized occupancy at that position.

### Introns and exons

Genome annotations for the WS190 *C*. *elegans* genome were obtained from the Ensembl project (http://www.ensembl.org). We restricted our analysis of exons and introns to those that met the following constraints: each intron must be at least 125 bp in length and each exon must be flanked on either side by introns passing this requirement. In addition, exons must be between 50 and 500 bp in length. Note that these requirements exclude the first and last exons of every gene.

### Gene expression data

Expression levels were obtained from SAGE data for Illumina N2 young adults produced at the Michael Smith Genome Sciences Centre by the Genome BC *C*. *elegans* Gene Expression Consortium (http://elegans.bcgsc.ca) [57]. We collated all SAGE tags with a valid match to a gene and ranked each gene according to how many times it was tagged. The top and bottom 10% of ranked genes constitute the strong and weak expression groups, respectively (genes without any tags were omitted).

### Splice site strength

The strength of a given splice site was calculated according to a position-specific weight matrix model [64,65]. If *w* is the DNA sequence of a splice site and *w*_*i*_ is the *i*’th nucleotide in that sequence, the strength *S*^*w*^ of the splice site is given by $S_{w}=\sum_{i}\log\left(p_{w_{i}}^{i}/b_{w}{}_{{}_{i}}\right)$, where $p_{w_{i}}^{i}$ is the probability to find the base *wi* at position *i* in a splice site, and $b_{w}{}_{{}_{i}}$ is the genome-wide frequency of *w*_*i*_. Two $p_{w_{i}}^{i}$ matrices were obtained by aligning all 3’ and 5’ splice sites respectively, and by computing how frequently base *w*_*i*_ appears at position *i*. 5’ (donor) splices sites are defined as containing 3 exonic and 7 intronic bases, while 3’ (acceptor) splice sites contain 3 exonic and 26 intronic bases [64,66]. The scores *S*_*w*_ for all 3’ and 5’ splice sites were tabulated and the top 10% of exons from either group were defined as “strong” splice sites, while the bottom 10% were defined as “weak” splice sites.

### Bioinformatics nucleosome positioning model

Our bioinformatics model is based on the position-independent component of the model developed by Kaplan et al. [26], although it uses dinucleotides instead of 5-mers and thus serves as the counterpart of the *N* = 2 position-independent model. For each dinucleotide *w*, we define the set *M*_*w*_ as the set of all positions *i* such that a nucleosome starting at *i* would cover the subsequence *w*, and we define *m*_*w*_^*i*^ as the number of appearances of *w* in the sequence covered by a nucleosome starting at *i* (as with our biophysical models, we exclude the leading and trailing 3 bases of the sequence each nucleosome covers). We then define μ_*w*_ as the reciprocal weighted average: $\mu_{w}={\left(\sum_{i\in M_{w}}m_{w}^{i}O_{i}/\sum_{i\in M_{w}}m_{w}^{i}\right)}^{-1}$, where *O*_*i*_ is the un-normalized read coverage at *i*. We further define the score *P*_*w*_ of a given word as *P*_*w*_ = *μ*_*w*_/∑_*w*'_*μ*_*w*'_. Finally, the “energy” *E*_*i*_ for a nucleosome starting at position *i* is given by $E_{i}=\sum_{j=i+3}^{i+143}\ln\;P_{w_{j}}$, where *w*_*j*_ is the dinucleotide starting at position *j*. We apply this formula to all bases in the genome and transform the resulting score profile into occupancy using the same recursive algorithm as in the biophysical models [22,53].

### Availability of supporting data

Our data, predictions and software are available on the Nucleosome Explorer website, http://nucleosome.rutgers.edu. *In vitro* sequence reads have been deposited into the Sequence Read Archive (http://www.ncbi.nlm.nih.gov/sra; accession number SRA050182).

## Competing interests

The authors declare no competing interests.

## Authors’ contributions

GL created computational models of nucleosome occupancy and energetics. DH participated in analyzing the nucleosome models. SMJ carried out *in vitro* reconstitutions of histones on *C*.*elegans* DNA and participated in drafting the manuscript. AVM participated in data analysis and drafted the manuscript. All authors read and approved the final manuscript.

## Supplementary Material

Additional file 1 Supplementary Figures and Tables. Click here for file
