# Supplementary material for: Global remodeling of nucleosome positions in C. elegans
Source: BMC Genomics. 2013 Apr 26;14:284. doi: 10.1186/1471-2164-14-284 (PMC3663828; doi:10.1186/1471-2164-14-284)
Supplement: Additional file 1 — Supplementary Figures and Tables. [file 1471-2164-14-284-S1.docx]

**Supplementary Material for:**

**Global remodeling of nucleosome positions in *C. elegans***

George Locke^1^, Devorah Haberman^2^, Steven M. Johnson^3^ and Alexandre V. Morozov^1,4^

^1^ Department of Physics and Astronomy and BioMaPS Institute for Quantitative Biology

Rutgers University, Piscataway, NJ 08854, USA

^2^ Department of Chemistry

Princeton University, Princeton, NJ 08544, USA

^3^ Department of Microbiology and Molecular Biology

Brigham Young University, Provo, UT 84602, USA

^4^ Corresponding author:

136 Frelinghuysen Road

Piscataway, NJ 08854

E-mail: [morozov@physics.rutgers.edu](mailto:morozov@physics.rutgers.edu)

Tel: (848) 445-1387; Fax: (732) 445-4320

**Supplementary Figures**

**
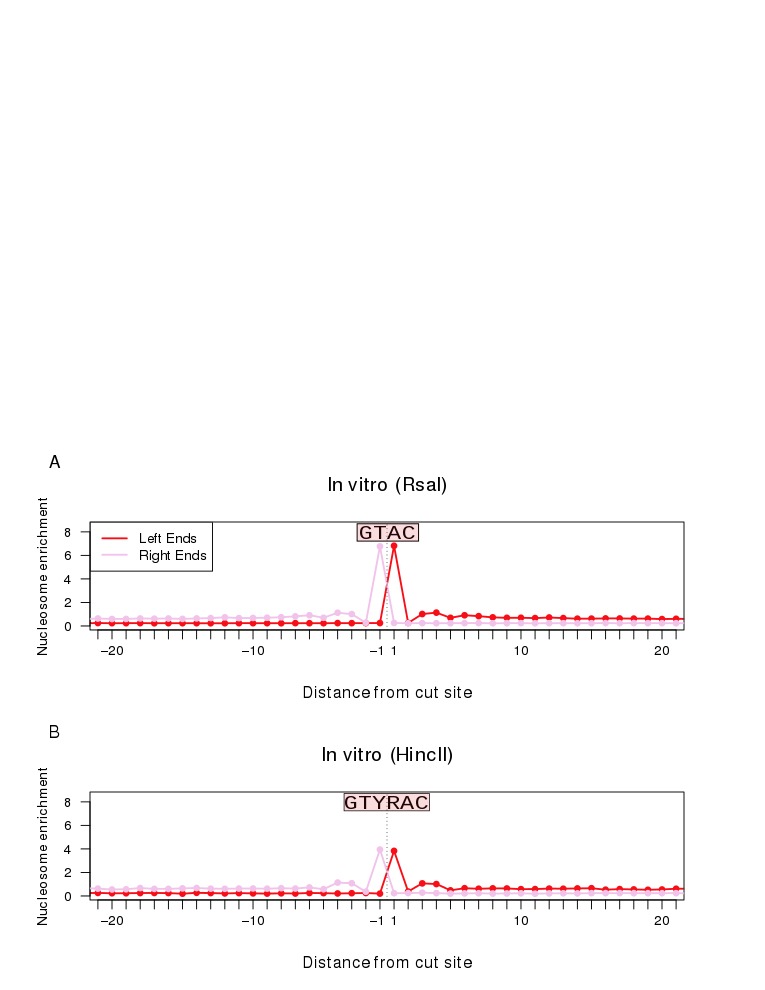
**

**Figure S1. Distribution of sequence reads observed *in vitro* in the vicinity of restriction enzyme cut sites. A)** Reads mapping to the positive strand indicate the presence of a
nucleosome to the right of the start of the read and thus represent the left end of a nucleosome. Reads mapping to the negative strand indicate the presence of a nucleosome to the left of
the start of the read and thus represent the right end of a nucleosome. For all Rsa I cut sites, the average number of left and right nucleosome ends from the Rsa I assay is plotted with
respect to the distance from these sites. The vertical scale is normalized to the genome-wide average number of reads per bp. **B)** Same as (A), but for the Hinc II dataset. Note that the peak at +1 is immediately to the right of the cut site, and the peak at -1 is immediately to the left. The cut site itself is in between the two base pairs.

**
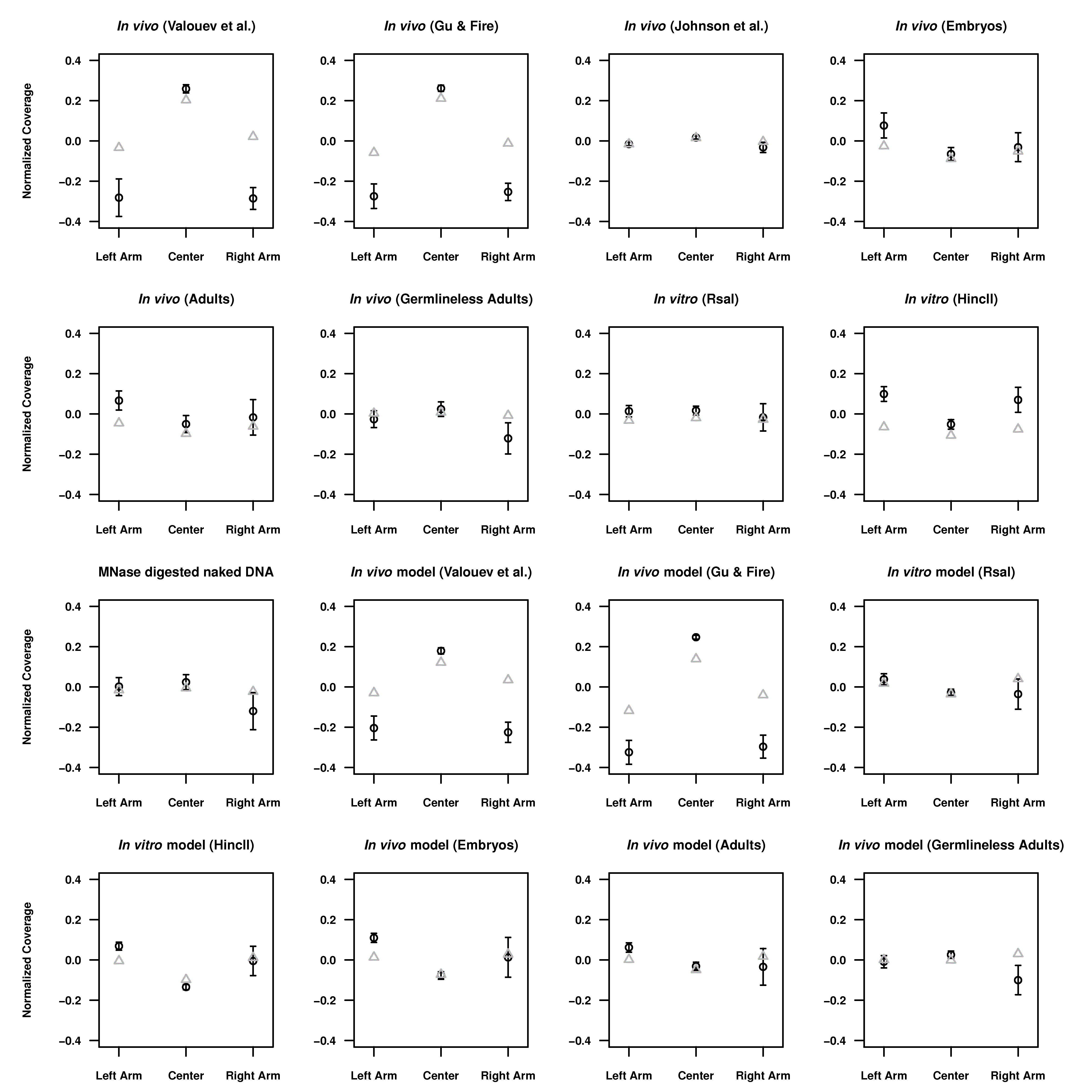
**

**Figure S2. Sequence read coverage in central and distal regions.** All autosomes were divided into three regions, with the left arm defined as the first 20%, the center as the middle 40%, and the right arm as the final 20% of the DNA sequence of each chromosome. For each of the three regions, the average read coverage normalized by the mean genome-wide read coverage was computed. The mean of these averages is shown as circles for the datasets indicated. Error bars are standard errors of the mean. Triangles show the average normalized read coverage for the X chromosome. The Johnson et al. *in vivo* nucleosome map is as previously described [[1](#_ENREF_1)]. Unlike all other datasets (**Materials and Methods**), the Johnson et al. data was not filtered for abnormally high and low nucleosome occupancy due to its very low read coverage. The restriction enzyme cut site filters (**Materials and Methods**) were not used for *in vitro* datasets.


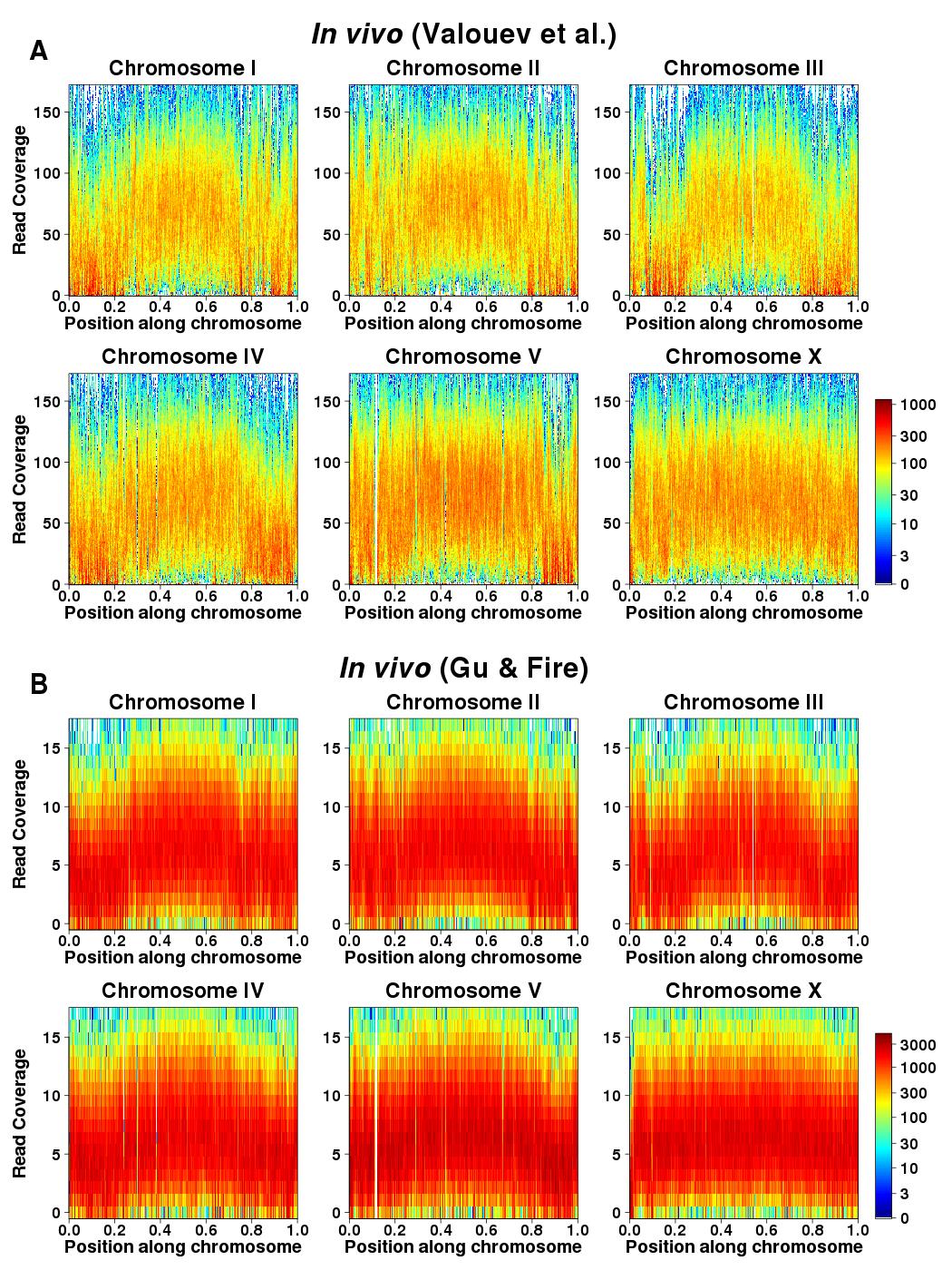


**Figure S3.** **Two-dimensional histograms of read coverage *in vivo***. **A)** Each *C. elegans* chromosome was divided into one thousand segments of equal length. The relative position of each segment is shown on the x-axis. The nucleosome read coverage, as measured *in vivo* by Valouev et al. [[2](#_ENREF_2)], is shown on the y-axis. For each segment, a color-coded histogram shows the number of bases with a given read coverage. The range of the y-axis excludes the top 1.0% of bases with the highest read coverage. **B)** Same as (A), with data from Gu & Fire [[3](#_ENREF_3)].


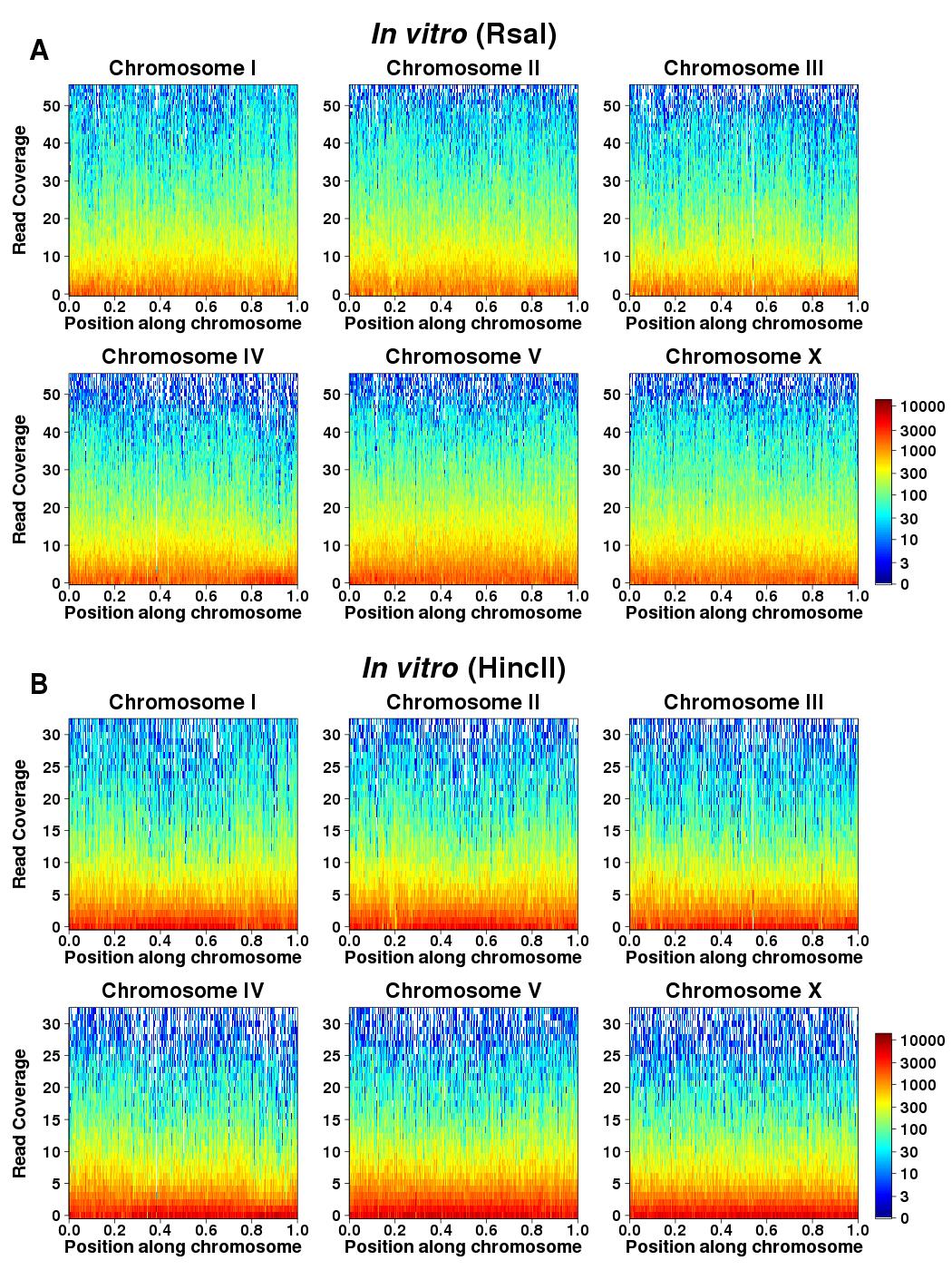


**Figure S4.** **Two-dimensional histograms of read coverage *in vitro***. **A)** Each *C. elegans* chromosome was divided into one thousand segments of equal length. The relative position of each segment is shown on the x-axis. The nucleosome read coverage, as measured by the Rsa I *in vitro* assay, is shown on the y-axis. For each segment, a color-coded histogram shows the number of bases with a given read coverage. The range of the y-axis excludes the top 1.0% of bases with the highest read coverage. **B)** Same as (A), with data from the Hinc II *in vitro* assay. The restriction enzyme cut site filters (**Materials and Methods**) were not employed.


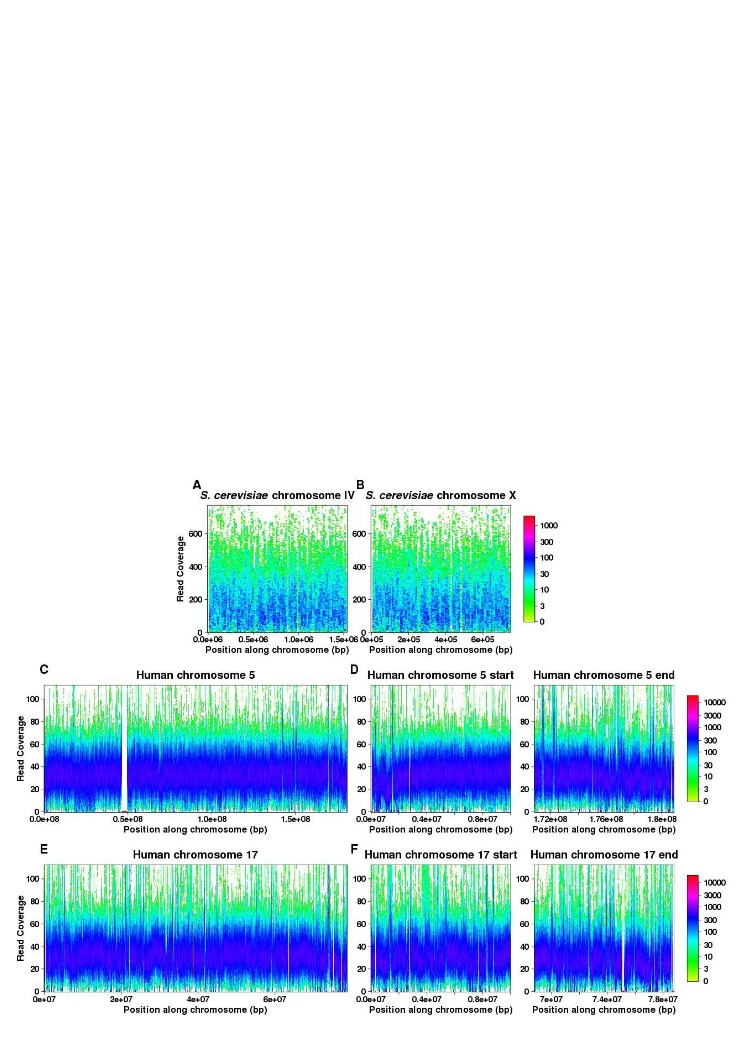


**Figure S5. Two-dimensional histograms of *in vivo* nucleosome read coverage in *S. cerevisiae* and *H. sapiens*.** **A)** *S. cerevisiae* chromosome IV was divided into one hundred segments of equal length. The relative position of each segment is shown on the x-axis. The nucleosome read coverage, as measured *in vivo* by Kaplan et al. (YPD assay without cross-linking, all replicates combined) [[4](#_ENREF_4)], is shown on the y-axis. For each segment, a color-coded histogram shows the number of bases with a given read coverage. The range of the y-axis excludes the top 1.0% of bases with the highest read coverage. **B)** Same as (A), but for *S. cerevisiae* chromosome X. **C)** Human chromosome 5 was divided into 20 kbp-long segments. The relative position of each segment is shown on the x-axis. The nucleosome read coverage, as measured *in vivo* by Valouev et al. (granulocyte assay) [[5](#_ENREF_5)], is shown on the y-axis. For each segment, a color-coded histogram shows the number of bases with a given read coverage. The range of the y-axis excludes the top 1.0% of bases with the highest read coverage. The data were not subject to high- and low-occupancy filtering. **D)** Same as (C), but zoomed in on the first and last 10 Mbp in chromosome 5. **E)** Same as (C), but for human chromosome 17. **F)** Same as (E), but zoomed in on the first and last 10 Mbp in chromosome 17.


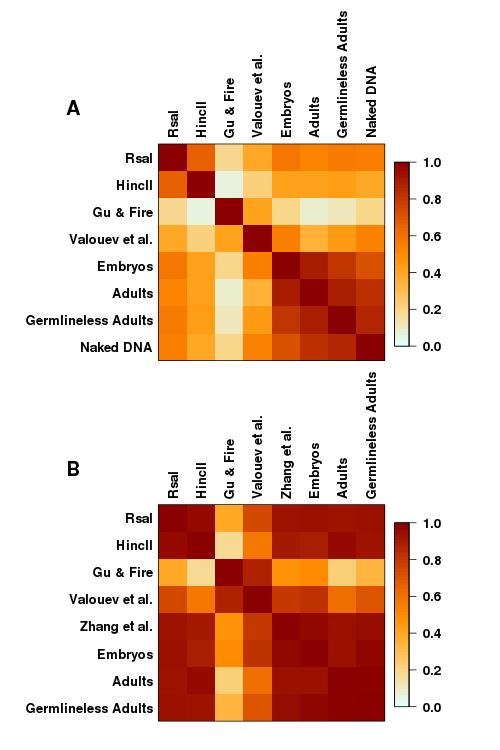


**Figure S6.** **A)** ***In vitro* and *in vivo* nucleosome maps**. Pearson correlations between read coverage profiles from each indicated experiment are plotted. **B) Position-independent models**. *N=2* position-independent (PI) models fit on the datasets indicated were used to predict nucleosome occupancy profiles on the *C. elegans* genome. Pearson correlations between predicted occupancy profiles are plotted. In (A) and (B), a single correlation coefficient is calculated across all chromosomes for each comparison.

**
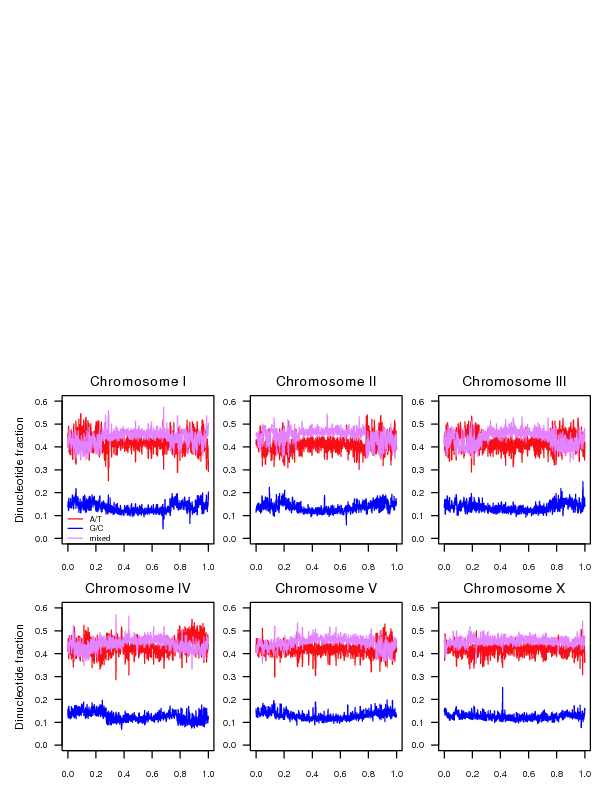
**

**Figure S7.** **Chromosome-wide** **dinucleotide frequency distributions**. Each *C. elegans* chromosome is divided into one thousand segments of equal length, and the dinucleotide composition in each segment is plotted against its relative genomic coordinate. A/T dinucleotides, composed only of A and T nucleotides, are shown in red, G/C dinucleotides, composed only of G and C nucleotides, are shown in blue, and mixed dinucleotides, including one A or T and one G or C nucleotide, are shown in pink.


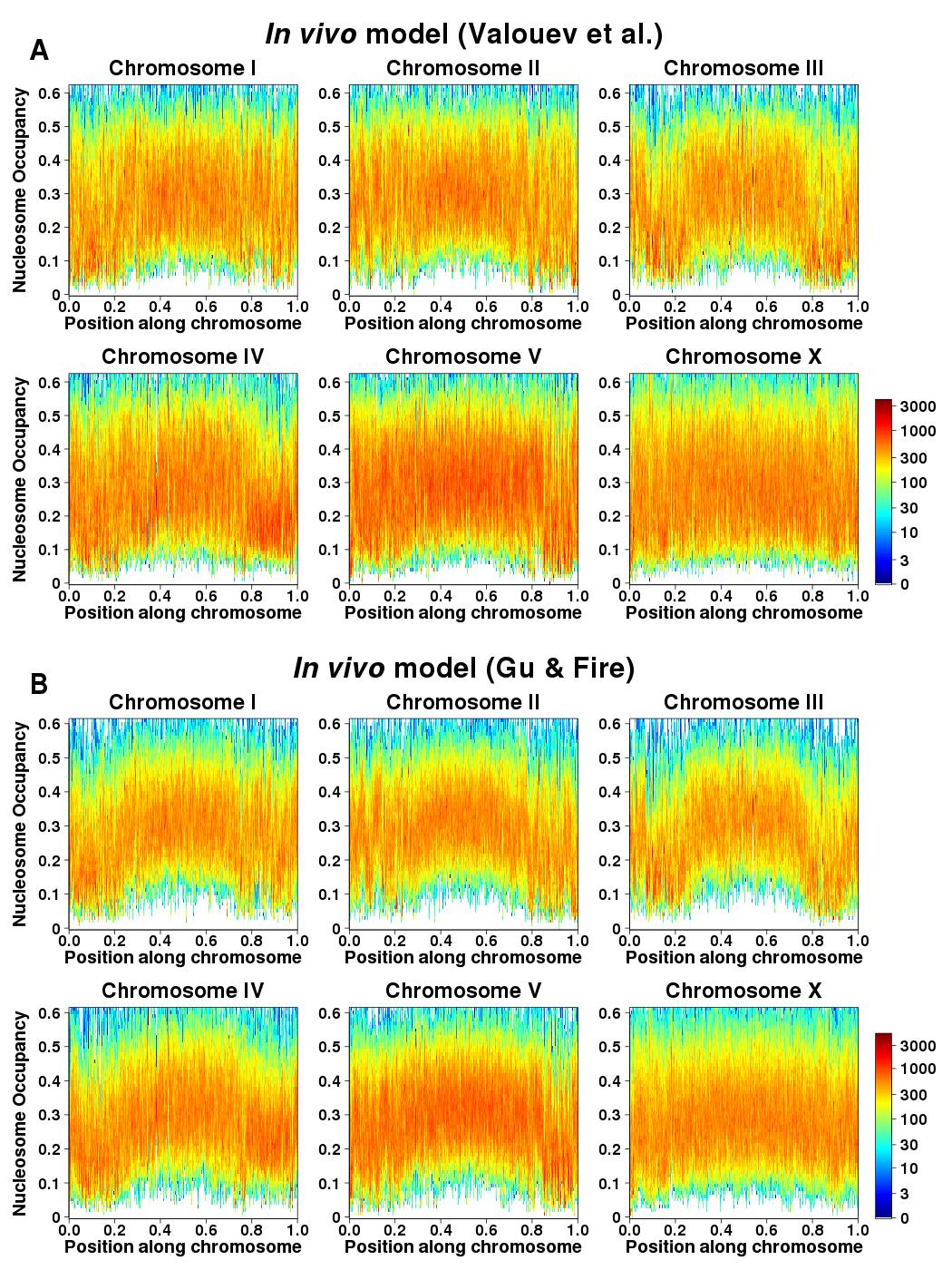


**Figure S8.** **Two-dimensional histograms of nucleosome occupancy predicted by position-independent *in vivo* models. A)** Each *C. elegans* chromosome was divided into one thousand segments of equal length. The relative position of each segment is shown on the x-axis. The nucleosome occupancy, as predicted by the *N=2* position-independent model fit on *in vivo* data from Valouev et al. [[2](#_ENREF_2)], is shown on the y-axis. For each segment, a color-coded histogram shows the number of bases with a given occupancy. The range of the y-axis excludes the top 1.0% of bases with the highest occupancy. **B)** Same as (A), but using occupancy predicted by the *N=2* position-independent model fit on *in vivo* data from Gu & Fire [[3](#_ENREF_3)].


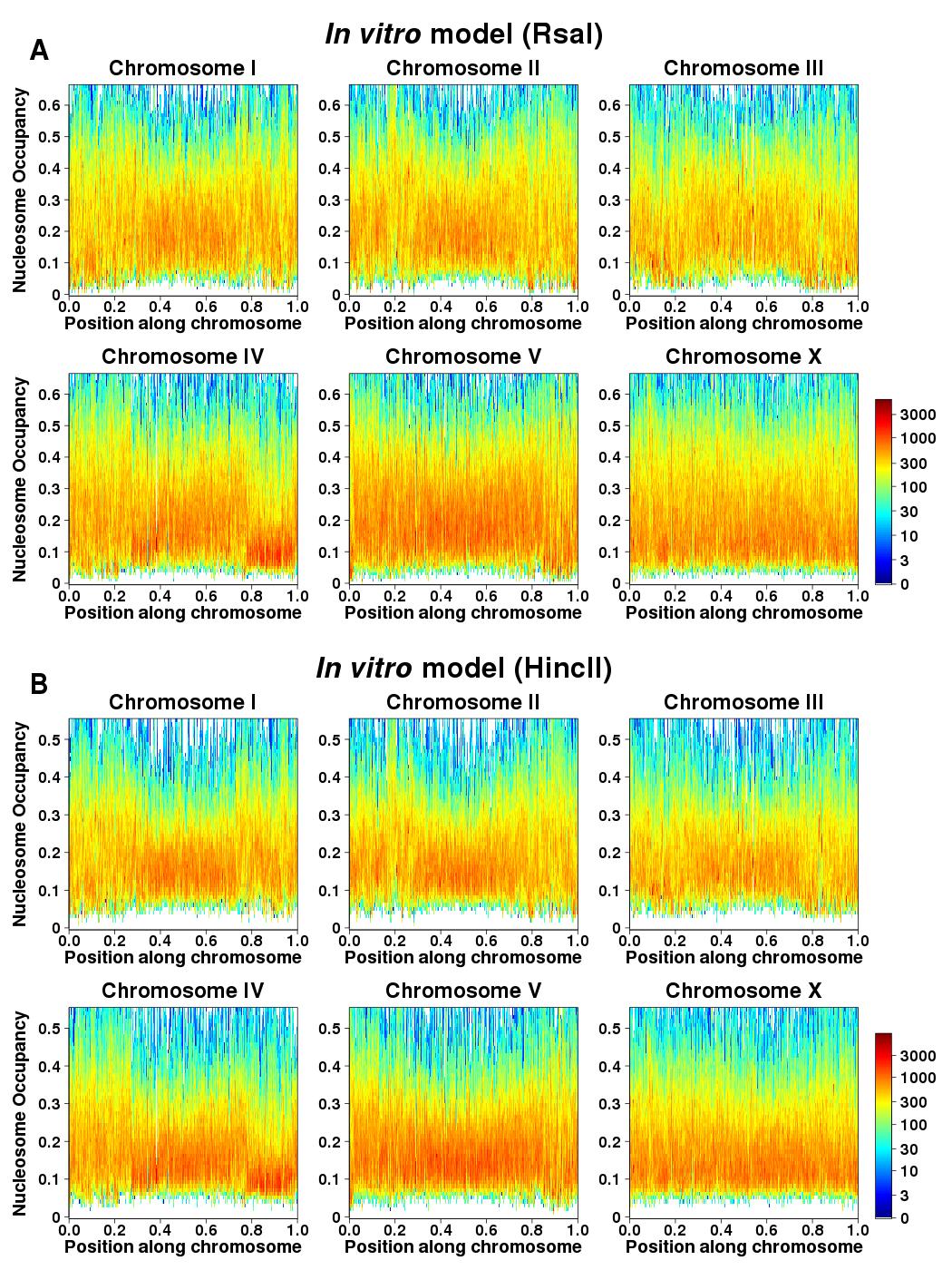


**Figure S9. Two-dimensional histograms of nucleosome occupancy predicted by position-independent *in vitro* models. A)** Each *C. elegans* chromosome was divided into one thousand segments of equal length. The relative position of each segment is shown on the x-axis. The nucleosome occupancy, as predicted by the *N=2* position-independent model fit on *in vitro* data from the Rsa I assay, is shown on the y-axis. For each segment, a color-coded histogram shows the number of bases with a given occupancy. The range of the y-axis excludes the top 1.0% of bases with the highest occupancy. **B)** Same as (A), but using occupancy predicted by the *N=2* position-independent model fit on *in vitro* data from the Hinc II assay.


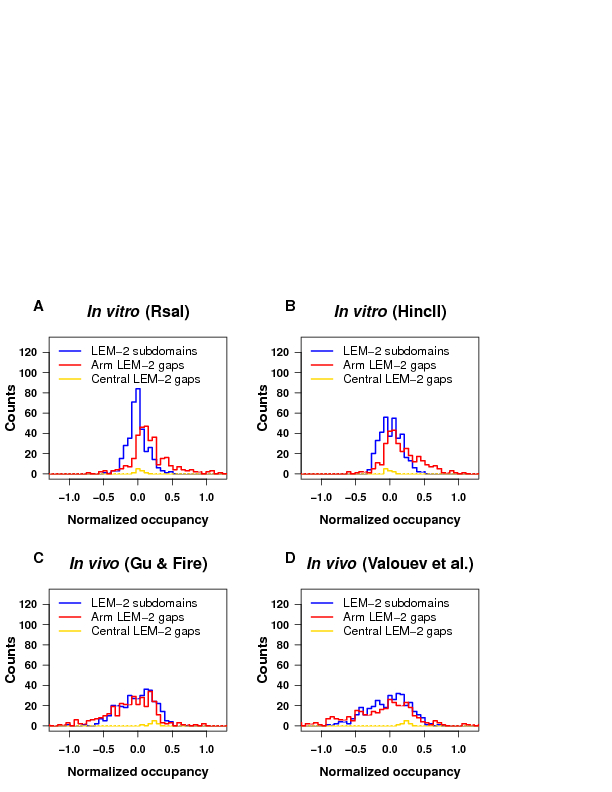


**Figure S10.** **Histograms of mean *in vitro* and *in vivo* nucleosome occupancies in LEM-2 gaps and subdomains. A)** Mean normalized nucleosome occupancy from the Rsa I *in vitro* assay is computed for LEM-2 gaps and sub-domains identified by Ikegami et al. [[6](#_ENREF_6)]. Gaps are further divided into those found on chromosomal arms (outside the interior 40% of each chromosome) and large central gaps. **B)** Same as (A), but using *in vitro* data from the Hinc II assay. **C)** Same as (A), but using *in vivo* data from Gu & Fire [[3](#_ENREF_3)]. **D)** Same as (A), but using *in vivo* data from Valouev et al. [[2](#_ENREF_2)].


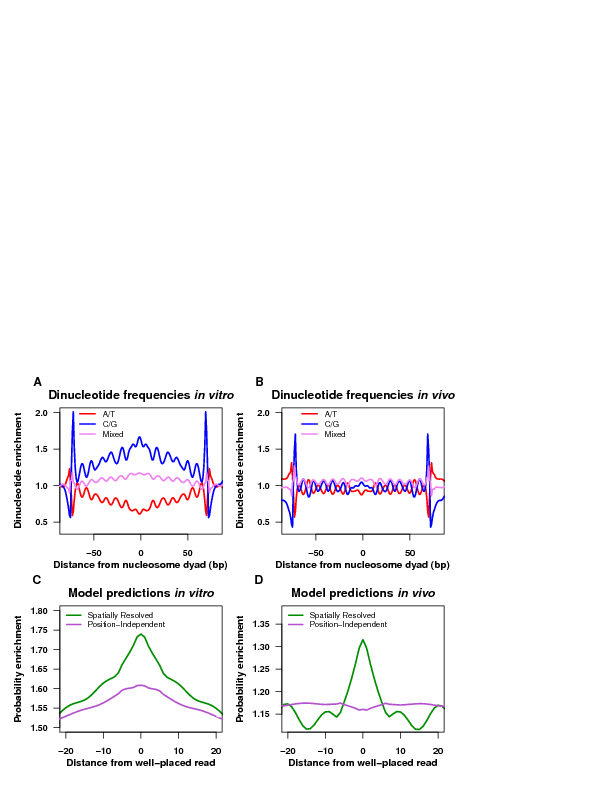


**Figure S11.** **A) Dinucleotide frequencies in well-positioned *in vitro* nucleosomes**. Each curve shows a relative dinucleotide frequency at a given position (with respect to the nucleosome dyad) for the set of well-placed nucleosomes selected from the Hinc II *in vitro* assay (see **Materials and Methods**). Dinucleotide counts used to calculate the frequencies include both forward and reverse strands for each well-placed nucleosome. We define the relative frequency of a group of dinucleotides as the sum of frequencies of all dinucleotides in that group at a given position, divided by the sum of genome-wide frequencies of the same dinucleotides. The groups plotted (with a 3-bp moving average) are AA/AT/TA/TT, CC/CG/GC/GG, and mixed dinucleotides (one A or T and one G or C nucleotide). **B) Dinucleotide frequencies in well-positioned *in vivo* nucleosomes**. Same as (A) but using well-placed nucleosomes from the Gu & Fire dataset [[3](#_ENREF_3)]. **C) Predicting well-positioned nucleosomes *in vitro***. Each curve shows probability enrichment predicted by a given model at a given distance from well-placed nucleosomes. Probability enrichment is defined as the predicted probability at a given position, divided by the genome-wide mean of the predicted probability profile. Probability enrichment is averaged over all well-placed nucleosomes in the Hinc II *in vitro* assay; the resulting curves are smoothed with a 7-bp moving average. The two models shown, *N=2* position-independent (magenta) and spatially resolved (green), were fit on the Hinc II *in vitro* data. **D) Predicting well-positioned nucleosomes *in vivo***. Same as (C) but with models fit on, and well-placed nucleosomes selected from the Gu & Fire dataset [[3](#_ENREF_3)].


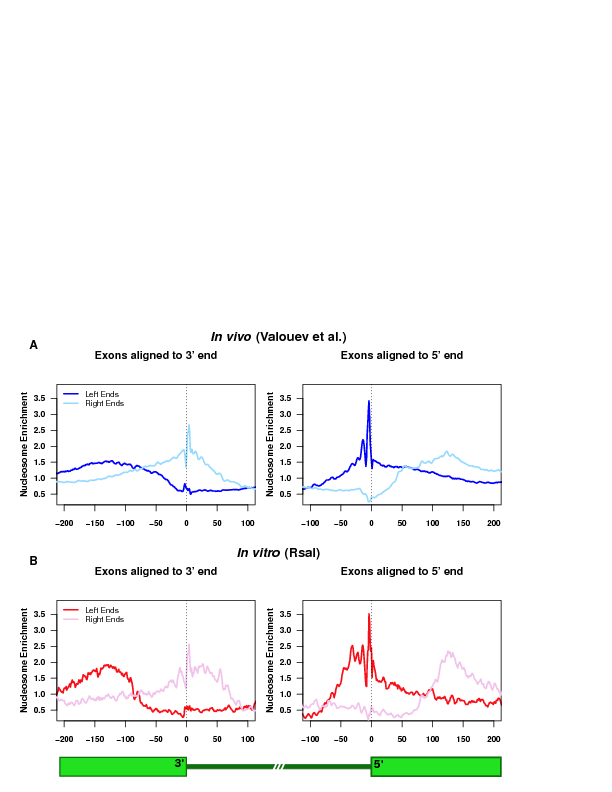


**Figure S12. Distribution of sequence reads in exon-intron regions.** Average number of sequence reads (which define left and right nucleosome ends as in **Fig. S1**) are plotted with respect to 3' and 5' exon-intron boundaries. Exons transcribed in both positive and negative directions are included. The blue curves (**A**) are based on the Valouev et al. *in vivo* dataset. The red curves (**B**) are based on the Rsa I *in vitro* dataset. The vertical scale is normalized to the genome-wide average number of reads per bp, and all curves are smoothed with a 5-bp running average.


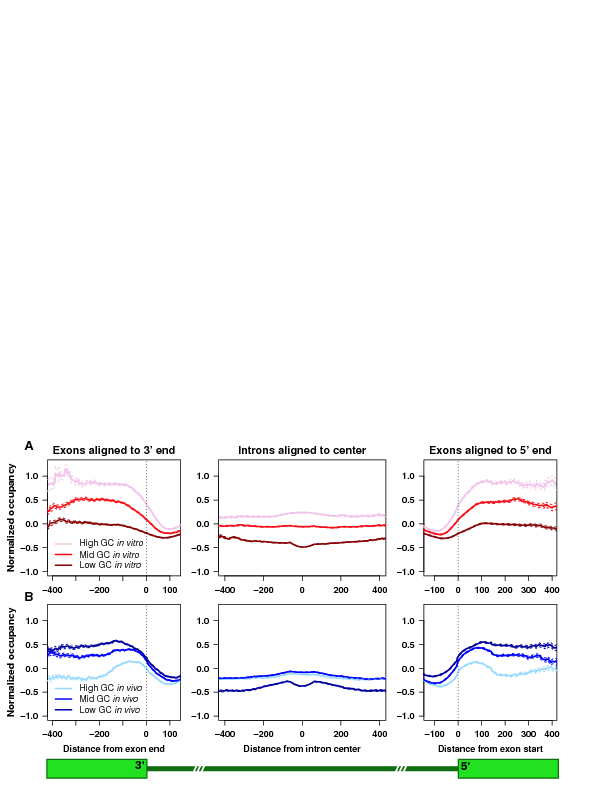


**Figure S13. *In vitro* and *in vivo* nucleosome occupancy in exons and introns grouped by GC content**. **A)** Exons and introns were divided into three equally sized groups of high, medium, and low GC content. Introns were aligned on their center, and exons were aligned to their 3’ ends (left) and 5’ ends (right). Mean normalized nucleosome occupancy of introns in each group is plotted against the distance from the intron center, and nucleosome occupancy of exons in each group is plotted against the distance from either the 3’ or the 5’ exon boundary. Averages *x* bases upstream of the 3’ boundary or downstream of the 5’ boundary are calculated only among exons of length ≥ *x*. The average intron occupancy a distance *x* from the intron center is calculated only among introns of length ≥ *2x*. Dashed curves show standard errors of the mean. The nucleosome occupancy profile is from the Hinc II *in vitro* assay. **B)** Same as (A), but using *in vivo* data from Gu & Fire [[3](#_ENREF_3)].

**
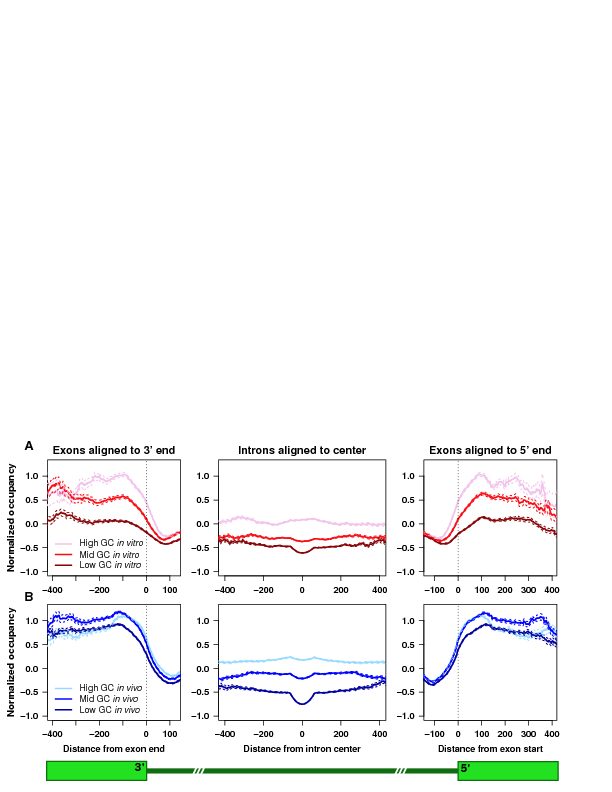
**

**Figure S14.** ***In vitro* and *in vivo* nucleosome occupancy in exons and introns located in chromosome central regions, grouped by GC content**. **A)** Exons and introns from the central 40% of each *C. elegans* chromosome were divided into three equally sized groups of high, medium, and low GC content. Introns were aligned on their center, and exons were aligned to their 3’ ends (left) and 5’ ends (right). Mean normalized nucleosome occupancy of introns in each group is plotted against the distance from the intron center, and nucleosome occupancy of exons in each group is plotted against the distance from either the 3’ or the 5’ exon boundary. Averages *x* bases upstream of the 3’ boundary or downstream of the 5’ boundary are calculated only among exons of length ≥ *x*. The average intron occupancy a distance *x* from the intron center is calculated only among introns of length ≥ *2x*. Dashed curves show standard errors of the mean. The nucleosome occupancy profile is from the Rsa I *in vitro* assay. **B)** Same as (A), but using *in vivo* data from Valouev et al. [[2](#_ENREF_2)].


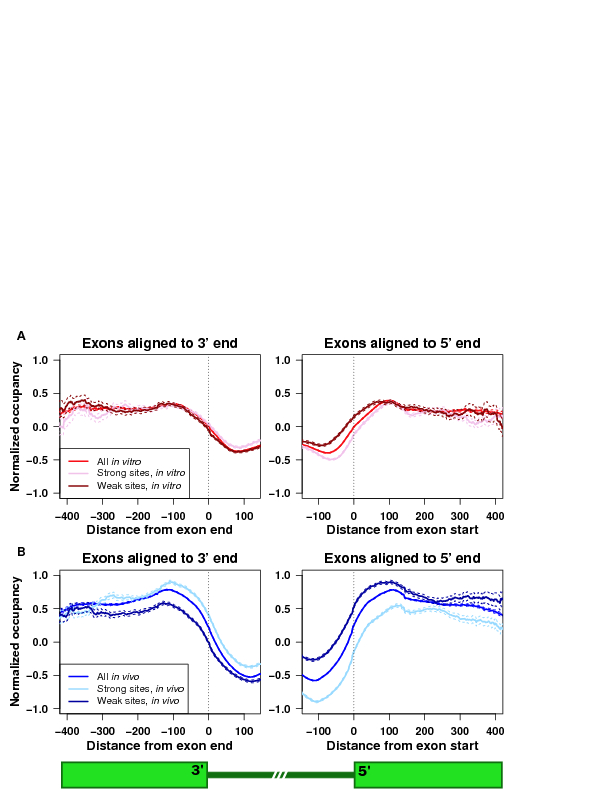


**Figure S15.** **A)** ***In vitro* nucleosome occupancy in exons grouped by splice site strength**. Exons are ranked according to the strength of their 5’ splice sites (left) and 3’ splice sites (right) (**Materials and Methods**). The pink curves show the average normalized nucleosome occupancy of exons with splice sites ranking in the top 10%, while the dark red curves show the average normalized nucleosome occupancy of exons in the bottom 10%. The bright red curves show nucleosome occupancy of all exons regardless of their splice site strength. Dashed curves show standard errors of the mean. Averages are calculated as in **Fig. S12** using data from the Rsa I assay. **B) *In vivo* nucleosome occupancy in exons grouped by splice site strength**. Same as (A), but using *in vivo* data from Valouev et al. [[2](#_ENREF_2)].


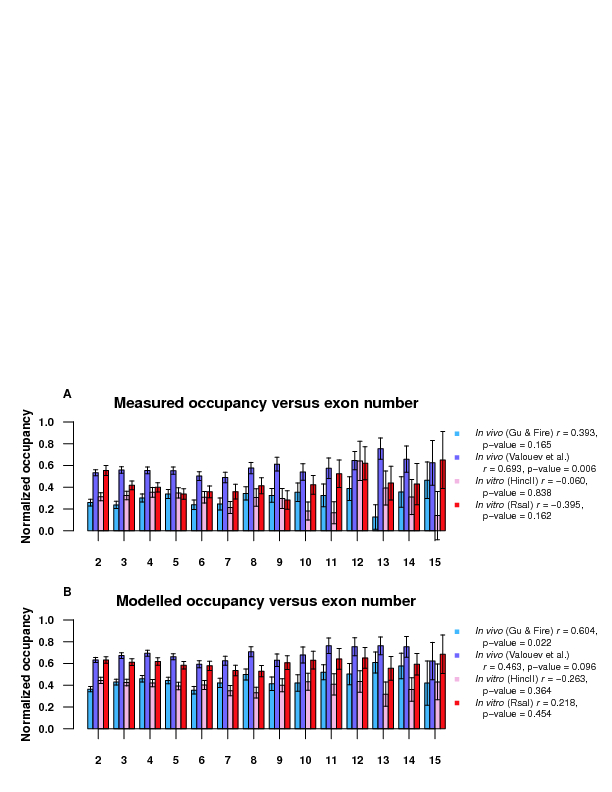


**Figure S16.** **Average exon nucleosome occupancy versus exon position within a gene**. The exons in each gene are numbered by their position in the gene. The average normalized nucleosome occupancy of each exon is calculated, and the means and standard errors of average nucleosome occupancy are plotted for each exon position. The legend shows the correlation coefficients between the plotted averages and exon number, as well as the p-value for each correlation. Genes containing less than four exons are excluded from the counts.

**Figure S17. A)** Agarose gel used to isolate invitrosome core DNA fragments from the Rsa I reconstitution. Lanes marked 1 and 5 are 1 µg/lane of the 1 Kb Plus DNA Ladder (Invitrogen), lane marked 3 is the MNase digested Rsa I invitrosome core DNA, and lanes marked 2 and 4 are 1µg of the 25 bp DNA Ladder (Invitrogen). In lanes 2 and 4 the bright band running just above the lowest band (100 bp) in lanes 1 and 5 is 125 bp, and the weaker band immediately above the 125 bp band is 150 bp. The top band (~150 bp) in lane 3 was excised and used for library preparation. Other bands in unmarked lanes are invitrosome isolations not used in this study. **B)** Agarose gel used to isolate invitrosome core DNA fragments from the Hinc II reconstitution. Lanes marked 1 and 4 are 1 µg/lane of the 25 bp DNA Ladder (Invitrogen). Lane marked 2 is 1 µg of the 1 Kb Plus DNA Ladder (Invitrogen), and lane marked 3 is the MNase digested Hinc II invitrosome core DNA. Ladder band sizes are as in (A). The top band (~150 bp) in lane 3 was excised and used for library preparation. **C)** Agarose gel used to assay Rsa I and Hinc II invitrosome libraries after completion of the Illumina Genomic DNA Sample Prep Kit procedure. Lanes marked 1 and 6 are 1µg of the 1 Kb Plus DNA Ladder (Invitrogen). Lanes marked 2 and 4 are 5 µl (out of 50 µl) of the Rsa I and Hinc II invitrosome libraries before the final PCR purification step, respectively. Lanes marked 3 and 5 are 3 µl (out of 30 µl) of the completed RsaI and Hinc II invitrosome libraries post PCR purification, respectively. Ladder band sizes are as in (A).


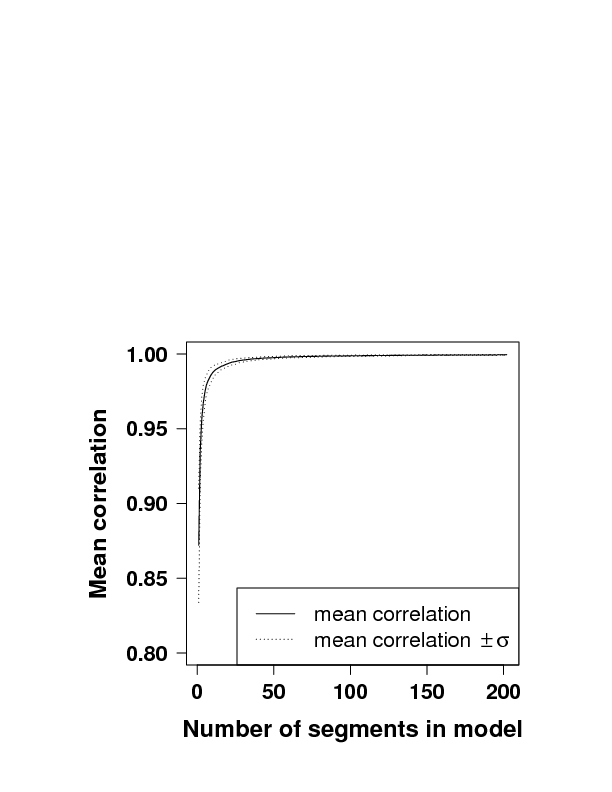


**Figure S18.** **Cross-validation of the spatially resolved model.** Genome-wide nucleosome energy profile based on the *in vivo* data from Gu & Fire [[3](#_ENREF_3)] was divided into 405 segments of equal size, and a spatially resolved model was fit separately to each segment. The 405 fits were divided into two groups of 202 (with one fit randomly omitted), and segments within each group were reshuffled. Using fits from each group, a sequence of averaged models was calculated: the averaged model at position *n* in the sequence was the average of first *n* models in the group. The correlations between all parameters (except $\varepsilon^{0}$) of the corresponding averaged models in the two groups were then calculated. The entire procedure was repeated 50 times. The solid curve shows the mean of 50 correlations calculated at each position $n$ in the sequence, while the dotted curves are one standard deviation away.

**Supplementary Tables**

**Table S1.** **Ranked dinucleotide energies predicted by position-independent models fit on chromosomal central regions**. The central regions were defined as the middle 40% of each chromosome. As in **Table 1**, dinucleotide energies $E_{w}$predicted by *N=2* position-independent models fit on the indicated datasets are shown ranked from highest (least favorable) to lowest (most favorable). The energy of a dinucleotide $w$ is defined as $E_{w}=\varepsilon_{w_{1}w_{2}}+\varepsilon_{w_{1}}+\varepsilon_{w_{2}},$ where $w_{1}$ and $w_{2}$ are the first and second nucleotides in $w$, and the $\varepsilon$’s are fitting parameters of the model (see **Materials and Methods**). Energy contributions are shown in arbitrary units, scaled so that each set of sixteen energies has zero mean and unit variance. A/T dinucleotides, composed only of A and T, are bolded, and G/C dinucleotides, composed only of G and C, are bolded and italicized.

| **Rank** | ***In vitro***  **(Rsa I)** | | ***In vitro***  **(Hinc II)** | | ***In vivo***  **(Gu et al.)** | | ***In vivo* (Valouev et al.)** | | ***In vivo (*Embryos)** | | ***In vivo* (Adults)** | | ***In vivo* (Germlineless Adults)** | |
| --- | --- | --- | --- | --- | --- | --- | --- | --- | --- | --- | --- | --- | --- | --- |
| 1 | **TT** | 1.49 | **AT** | 1.40 | ***GC*** | 1.81 | **TT** | 1.91 | **TA** | 1.35 | **TA** | 1.52 | **TA** | 1.61 |
| 2 | **AA** | 1.49 | **TT** | 1.39 | ***GG*** | 1.48 | **AA** | 1.91 | **TT** | 1.26 | **TT** | 1.35 | **TT** | 1.52 |
| 3 | **AT** | 1.36 | **AA** | 1.39 | ***CC*** | 1.48 | **TA** | 1.14 | **AA** | 1.26 | **AA** | 1.35 | **AA** | 1.52 |
| 4 | **TA** | 0.93 | **TA** | 0.74 | ***CG*** | 1.48 | ***CG*** | 0.77 | **AT** | 1.15 | **AT** | 1.12 | **AT** | 1.14 |
| 5 | TC | 0.29 | GA | 0.38 | **TT** | 0.48 | ***GC*** | 0.49 | CT | 0.32 | AG | 0.31 | CT | 0.23 |
| 6 | GA | 0.29 | TC | 0.38 | **AA** | 0.48 | **AT** | 0.45 | AG | 0.32 | CT | 0.31 | AG | 0.23 |
| 7 | AC | 0.23 | TG | 0.21 | **TA** | -0.20 | ***CC*** | 0.02 | TC | 0.11 | GA | 0.00 | TC | -0.08 |
| 8 | GT | 0.23 | CA | 0.21 | CT | -0.54 | ***GG*** | 0.02 | GA | 0.11 | TC | 0.00 | GA | -0.08 |
| 9 | CA | 0.00 | AC | 0.12 | AG | -0.54 | AG | -0.51 | AC | -0.04 | GT | -0.11 | AC | -0.29 |
| 10 | TG | 0.00 | GT | 0.12 | **AT** | -0.67 | CT | -0.51 | GT | -0.04 | AC | -0.11 | GT | -0.29 |
| 11 | CT | -0.37 | CT | -0.20 | AC | -0.81 | GA | -0.71 | CA | -0.04 | TG | -0.20 | CA | -0.45 |
| 12 | AG | -0.37 | AG | -0.20 | GT | -0.81 | TC | -0.71 | TG | -0.04 | CA | -0.20 | TG | -0.45 |
| 13 | ***CG*** | -1.16 | ***CC*** | -1.24 | TG | -0.88 | GT | -0.83 | ***GG*** | -1.16 | ***CC*** | -1.05 | ***GG*** | -0.80 |
| 14 | ***GG*** | -1.17 | ***GG*** | -1.24 | CA | -0.88 | AC | -0.83 | ***CC*** | -1.16 | ***GG*** | -1.05 | ***CC*** | -0.80 |
| 15 | ***CC*** | -1.17 | ***CG*** | -1.48 | GA | -0.95 | TG | -1.31 | ***GC*** | -1.59 | ***GC*** | -1.52 | ***GC*** | -1.43 |
| 16 | ***GC*** | -2.05 | ***GC*** | -1.97 | TC | -0.95 | CA | -1.31 | ***CG*** | -1.80 | ***CG*** | -1.74 | ***CG*** | -1.58 |

**References**

1. Johnson SM, Tan FJ, McCullough HL, Riordan DP, Fire AZ: **Flexibility and constraint in the nucleosome core landscape of Caenorhabditis elegans chromatin.** *Genome Research* 2006, **16:**1505-1516.

2. Valouev A, Ichikawa J, Tonthat T, Stuart J, Ranade S, Peckham H, Zeng K, Malek JA, Costa G, McKernan K, et al: **A high-resolution, nucleosome position map of C. elegans reveals a lack of universal sequence-dictated positioning.** *Genome Research* 2008, **18:**1051-1063.

3. Gu SG, Fire A: **Partitioning the C. elegans genome by nucleosome modification, occupancy, and positioning.** *Chromosoma* 2010, **119:**73-87.

4. Kaplan N, Moore IK, Fondufe-Mittendorf Y, Gossett AJ, Tillo D, Field Y, LeProust EM, Hughes TR, Lieb JD, Widom J, Segal E: **The DNA-encoded nucleosome organization of a eukaryotic genome.** *Nature* 2009, **458:**362-366.

5. Valouev A, Johnson SM, Boyd SD, Smith CL, Fire AZ, Sidow A: **Determinants of nucleosome organization in primary human cells.** *Nature* 2011, **474:**516-520.

6. Ikegami K, Egelhofer TA, Strome S, Lieb JD: **Caenorhabditis elegans chromosome arms are anchored to the nuclear membrane via discontinuous association with LEM-2.** *Genome Biology* 2010, **11:**R120.
